# Na-Promoted Bimetallic Hydroxide Nanoparticles for Aerobic C–H Activation: Catalyst Design Principles and Insights into Reaction Mechanism

**DOI:** 10.1021/acsami.4c11070

**Published:** 2024-10-25

**Authors:** Beyzanur Erdivan, Eylul Calikyilmaz, Suay Bilgin, Ayse Dilay Erdali, Damla Nur Gul, Kerem Emre Ercan, Yunus Emre Türkmen, Emrah Ozensoy

**Affiliations:** † Department of Chemistry, Faculty of Science, Bilkent University, 06800 Ankara, Türkiye; ‡ Roketsan Inc., Elmadag, 06780 Ankara, Türkiye; § UNAM - National Nanotechnology Research Center and Institute of Materials Science and Nanotechnology, Bilkent University, 06800 Ankara, Türkiye

**Keywords:** alkylarene C−H activation, C−H oxidation, bimetallic hydroxide catalysts, heterogeneous catalysis, catalytic alkali promotion

## Abstract

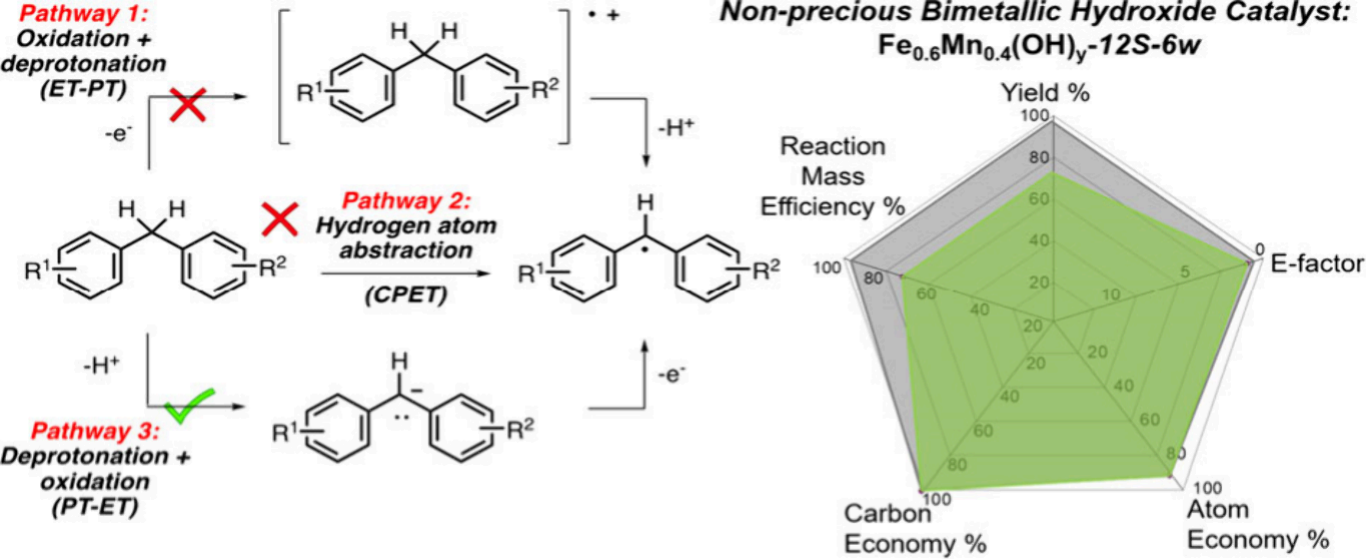

A precious metal-free bimetallic Fe_*x*_Mn_1–*x*_(OH)_*y*_ hydroxide catalyst was developed that is capable of catalyzing aerobic C–H oxidation reactions at low temperatures, without the need for an initiator, relying sustainably on molecular oxygen. Through a systematic synthetic effort, we scanned a wide nanoparticle synthesis parameter space to lay out a detailed set of catalyst design principles unraveling how the Fe/Mn cation ratio, NaOH(aq) concentration used in the synthesis, catalyst washing procedures, extent of residual Na^+^ promoters on the catalyst surface, reaction temperature, and catalyst loading influence catalytic C–H activation performance as a function of the electronic, surface chemical, and crystal structure of Fe_*x*_Mn_1–*x*_(OH)_*y*_ bimetallic hydroxide nanostructures. Our comprehensive XRD, XPS, BET, ICP-MS, ^1^H NMR, and XANES structural/product characterization results as well as mechanistic kinetic isotope effect (KIE) studies provided the following valuable insights into the molecular level origins of the catalytic performance of the bimetallic Fe_*x*_Mn_1–*x*_(OH)_*y*_ hydroxide nanostructures: (i) catalytic reactivity is due to the coexistence and synergistic operation of Fe^3+^ and Mn^3+^ cationic sites (with minor contributions from Fe^2+^ and Mn^2+^ sites) on the catalyst surface, where in the absence of one of these synergistic sites (i.e., in the presence of monometallic hydroxides), catalytic activity almost entirely vanishes, (ii) residual Na^+^ species on the catalyst surface act as efficient electronic promoters by increasing the electron density on the Fe^3+^ and Mn^3+^ cationic sites, which in turn, presumably enhance the electrophilic adsorption of organic reactants and strengthen the interaction between molecular oxygen and the catalyst surface, (iii) in the fluorene oxidation reaction the step dictating the reaction rate likely involved the breaking of a C–H bond (*k*_H_*/k*_D_ = 2.4), (iv) reactivity patterns of a variety of alkylarene substrates indicate that the C–H bond cleavage follows a stepwise PT-ET (proton transfer-electron transfer) pathway.

## 1 Introduction

Organic synthesis relies on converting existing functional groups into others through catalytic or noncatalytic methods. In this respect, besides C–C bond-forming reactions, functional group interconversions constitute the most commonly employed strategy in organic synthesis, which involves transformation of pre-existing functional groups, often requiring multiple steps. In recent decades, there has been a significant interest in catalytically functionalizing previously unactivated (sp^2^ and sp^3^) C–H bonds across different fields of chemistry. C–H activation reactions are significant for producing valuable products, such as alcohols or carbonyl-containing groups. Such transformations are economically efficient, occurring in a single step without prior functionalization. However, catalyzing the activation of C–H bonds is challenging due to their high bond dissociation energies and the nonpolar nature of these bonds.^1−4^ A crucial subclass of C–H activation reactions is C–H oxidation reactions.^5−8^ Heterogeneous catalytic systems for some C–H oxidation reactions lag behind homogeneous counterparts in reactivity and selectivity.^9−11^ Yet, catalytic approaches using nonprecious, abundant transition metals as catalysts and molecular oxygen as the stoichiometric oxidant are environmentally and economically appealing. Therefore, there is a high demand for heterogeneous oxidation catalysts composed of nonprecious metals, functioning under mild conditions.

Various metal oxide systems, serving as heterogeneous catalysts, exhibit high-temperature efficiency but often face limitations in low-temperature catalytic activity, particularly in the absence of environmentally harmful and aggressive oxidants.^12^ Thus, the development of platinum group metal (PGM)-free heterogeneous catalytic systems that can be activated at low temperatures via mild oxidizing agents is pivotal for cost reduction and sustainability. Metal hydroxide systems, composed of nonprecious transition metals, offer a notable advantage with multiple valence states, allowing a broad range of redox chemistry. One of the unique features of the metal hydroxide-based catalyst systems lies in their ability to possess both Lewis acidic and Brønsted basic active sites.^13^ This dual functionality may activate various substrates (i.e., adsorbates) through the cooperative action of Lewis acid and Brønsted base pair sites.^13^ Metal hydroxides have been extensively employed in various fields including battery technologies, electrocatalysis, electrosynthesis, photocatalysis, supercapacitors, electrochromic devices, and electrochemical sensors.^14^ Previous studies have demonstrated that some of these catalysts facilitate reactions at considerably lower temperatures compared to noble metal-based catalysts.^12^ Mixed-transition metal catalysts, incorporating more than one type of cation, exhibit favorable catalytic behavior through the cooperation of multiple active sites, synergistic electronic effects due to the presence of multiple oxidation states, different d-band structures, as well as unique geometric, morphological, and crystallographic properties.^15^ Continuing our efforts to develop effective heterogeneous catalysts for alcohol and C–H bond oxidation reactions under aerobic conditions, we recently reported the use of LaMnO_3_ as a perovskite-based catalyst and PGM-free 2D mixed metal layered double hydroxide (LDH) systems for alkylarene and alcohol oxidation reactions as well as oxidative dimerization of 2-naphthol,^16,17^ in line with other parallel efforts reported in the literature utilizing different heterogeneous catalytic architectures.^18−21^

Recent investigations have highlighted the positive impact of alkali metal doping in various catalytic systems.^22−25^ Alkali metal ions, such as Na^+^, have shown the potential to act as electronic promoters by reducing the oxidation states of metal catalysts in diverse catalytic processes. For example, a former report demonstrated the favorable effect of Cs^+^ and Na^+^ additions on the activity of Mn_2_O_3_ in the catalytic combustion of ketones.^24^ They observed that the presence of alkali metal additives increased the electron density of the Mn_2_O_3_ surface, enhancing the electrophilic adsorption of ketone molecules. Another study revealed that surface Na species increased the adsorption energy of O_2_ during CO oxidation at low temperatures, resulting in a decreased energy barrier for the transition states formed on the bimetallic NaAu_2_ catalysts.^23^

Remarkably, iron is the most abundant transition metal and the fourth most abundant element in Earth’s crust, whereas manganese is the third most abundant transition metal.^26,27^ Such high natural abundances of these two transition metals coupled with their low cost and ability to acquire a broad range of oxidation states make them ideal candidates to be used in sustainable and low-cost oxidation catalysts. In both homogeneous and heterogeneous systems, iron and manganese have been individually studied for C–H activation reactions.^18−21,28,29^ Here, in our current report, we demonstrate how precious metal-free Fe and Mn hydroxide systems, optimized through specific synthetic parameters, can serve as highly active and selective catalysts for low-temperature C–H activation reactions, using only molecular oxygen as an alternative green oxidant, with the noteworthy enhancement of performance through the introduction of Na^+^ acting as an electronic promoter.

## 2 Experimental Section

### 2.1 Catalyst Synthesis and Optimization Parameters

All chemicals, namely, Fe(NO_3_)_3_.9H_2_O (≥98% purity), Mn(NO_3_)_2_·4H_2_O (≥97% purity), and NaOH (≥98% purity, solubility in water: 1260 g/L at 20 °C), purchased from Sigma-Aldrich, were used as received in the catalyst synthesis. Assuming the presence of divalent Mn and trivalent Fe cations, the nominal stoichiometric concentration of NaOH(aq) required for the synthesis of a mixed metal hydroxide with equimolar Fe and Mn cations (i.e., Fe_0.5_Mn_0.5_(OH)_2.5_) is 2.06 M. Hereafter, this stoichiometric hydroxide concentration will be represented as “*S*”, and the catalysts will be designated as Fe_*x*_Mn_*1–x*_(OH)_*y*_-*nS*, where *nS* (1 ≤ *n* ≤ 15) indicates the NaOH(aq) concentration used in the catalyst synthesis.

In the coprecipitation synthesis method utilized in the current work (Figure S1), calculated amounts of Fe(NO_3_)_3_·9H_2_O and Mn(NO_3_)_2_·4H_2_O (Table S1) were dissolved in 20 mL of deionized water. In a separate beaker, an appropriate amount of NaOH (Table S2) was dissolved in 20 mL of deionized water. These solutions were stirred at 600 and 400 rpm, respectively. Before mixing, the stirring rate of NaOH(aq) was increased to 1500 rpm and the solution containing the metal precursors was added to the NaOH(aq) solution. The mixture was stirred at this high stirring rate for 1 min, and thereafter, the solution was left to mix for an additional hour at 600 rpm. Then, the mixture was washed with deionized water 6 times for 3 min at a rate of 6000 rpm in 4 separate 15 mL centrifuge tubes followed by drying in a 400 mL beaker at 60 °C for 24 h to obtain powder catalyst samples.

#### 2.1.1 Nominal Metal Cation Ratio Optimization in the Chemical Precipitation Method

For the synthesis of the optimized Fe–Mn hydroxide catalyst, the nominal Fe/Mn ratio was varied while maintaining a constant and a relatively high NaOH(aq) concentration of *12S* to ensure efficient hydroxylation of the catalysts with diverse Fe and Mn cation loadings. This set of catalysts was washed with deionized water 6 times at the end of the synthesis.

#### 2.1.2 NaOH Concentration Optimization in the Chemical Precipitation Method

Nominal metal cation mole optimization experiments revealed that the Fe_0.6_Mn_0.4_(OH)_*y*_ catalyst outperformed other currently tested relative metal loadings, exhibiting the highest catalytic activity in the aerobic oxidation of fluorene to fluorenone. Keeping this metal cation ratio constant, synthesis of the catalyst was carried out with different NaOH(aq) concentrations of *1S, 3S, 6S, 9S, 12S*, and *15S*. Note that *15S* is the saturation concentration of NaOH in water at room temperature. Catalysts were washed with deionized water 6 times at the end of the synthesis.

#### 2.1.3 Optimization of the Number of Washing Cycles in the Chemical Precipitation Method

To study the impact of residual Na^+^ ions on the catalytic aerobic oxidation of fluorene to fluorenone, Fe_0.6_Mn_0.4_(OH)_*y*_-*12S* catalyst was synthesized with varying numbers of subsequent washing cycles using deionized water (i.e., *n* = 1, 3, 6, and 9 times which are denoted as *nw* in the catalyst naming, Fe_0.6_Mn_0.4_(OH)_*y*_-*12S-6w*). It is worth emphasizing that washing the catalyst only once or three times proved to be insufficient to obtain catalysts with a measurable catalytic activity due to the formation of bulk Na salts on the catalyst surface.

#### 2.1.4 Influence of the Catalyst Amount and the Reaction Temperature

We also examined the influence of the catalyst amount (loading) on the fluorene oxidation reaction by utilizing 10, 15, and 20 mg of the optimized Fe_0.6_Mn_0.4_(OH)_*y*_-*12S-6w* catalyst. Furthermore, fluorene oxidation reaction was also carried out at 60, 80, and 90 °C to investigate the impact of temperature on catalytic activity.

#### 2.1.5 Influence of the Type of the Alkali Metal Hydroxide Used in the Chemical Precipitation Method

To investigate the effect of the type of alkali metal hydroxide used in the synthesis, the synthesis of a Fe_0.6_Mn_0.4_(OH)_*y*_-*12S-6w* catalyst was also carried out with *12S* KOH (aq).

### 2.2 Catalytic Performance Tests

For liquid phase catalytic activity tests, an appropriate amount of catalyst and 2.0 mL of *n*-heptane as a solvent were loaded into an oven-dried 25 mL Schlenk flask. The flask was then filled with 1 bar of O_2_(g) and sealed with a stopcock. For anaerobic control experiments, the solvent was deoxygenated through the freeze–pump–thaw technique (five cycles) and N_2_(g) served as the inert medium. Unless otherwise mentioned, *n*-heptane was used as the solvent in all catalytic performance tests. In an oil bath, the reaction mixture was stirred at 400 rpm, at various temperatures for 24 h. After a typical catalytic performance test reaction, the mixture was cooled to room temperature (RT), diluted with ethyl acetate (EtOAc), and passed through Celite. Conversion values for catalytic oxidation reactions were determined via ^1^H NMR spectroscopic analysis. Flash column chromatography with Silicycle 40–63 μm (230–400 mesh) silica gel as the stationary phase was employed for purification.

### 2.3 Instrumentation

X-ray diffraction (XRD) experiments were conducted with a PANalytical (X’Pert PRO) Multi-Purpose X-ray diffractometer equipped with a Cu Kα (1.5405 Å) X-ray source operated at 45 kV/40 mA. Powder samples were analyzed within a 2θ range of 10–80° using a scan step size of 0.01° and a time per step of 35 s. The specific surface area (SSA) values of the catalysts were measured with a Micromeritics Tristar 3000 surface area and pore size analyzer via the 5-point Brunauer, Emmett, and Teller (BET) N_2_ adsorption method. Before SSA measurements, samples were outgassed at 130 °C overnight, and measurements were taken at −196 °C. A Thermo Scientific K-alpha spectrometer equipped with an Al K_α_ micro monochromatic source (1486.6 eV) was used to record the X-ray photoelectron spectroscopy (XPS) data. The survey XP spectra were recorded with 2 scans with a pass energy of 200 eV, while high-resolution XP spectra were acquired with averaging 20 scans with a pass energy of 30 eV. XPS data were analyzed using CasaXPS software, and binding energy (B.E.) values were adjusted using the C 1s signal of adventitious carbon at 284.8 eV. Agilent 7700x Inductively Coupled Plasma Mass Spectrometer (ICP-MS) was used to measure bulk elemental compositions. ICP-MS calibration solutions were prepared with standard solutions of Fe and Mn separately in 2% w/w HNO_3_(aq). X-ray Absorption Near Edge Spectroscopy (XANES) measurements were carried out at the BM08 XAFS/XRF (X-ray Absorption Fine Structure/X-ray Fluorescence) beamline of SESAME (Synchrotron-Light for Experimental Science and Applications in the Middle East, located in Allan, Jordan). XANES experiments were performed in absorption mode with 3 scans per sample. XANES energy calibration was done using metallic Fe and Mn foils as well as Fe(NO_3_)_3_ and Mn(NO_3_)_2_ powders. Analysis of the recorded XANES spectra was executed by using Athena software. ^1^H NMR (400 MHz) and ^13^C{^1^H}-NMR (100 MHz) spectra were recorded on a Bruker DPX 400 NMR spectrometer. CDCl_3_ was used as a solvent in the NMR experiments. The NMR data were calibrated against either the signal of an internal standard (TMS, tetramethylsilane, 0 ppm) or residual solvent signals (chloroform; 7.26 ppm in ^1^H NMR and 77.16 ppm in ^13^C NMR spectra).

## 3 Results and Discussion

### 3.1 Catalytic Performance Tests for the Oxidation of Fluorene to Fluorenone

**Figure 1:**
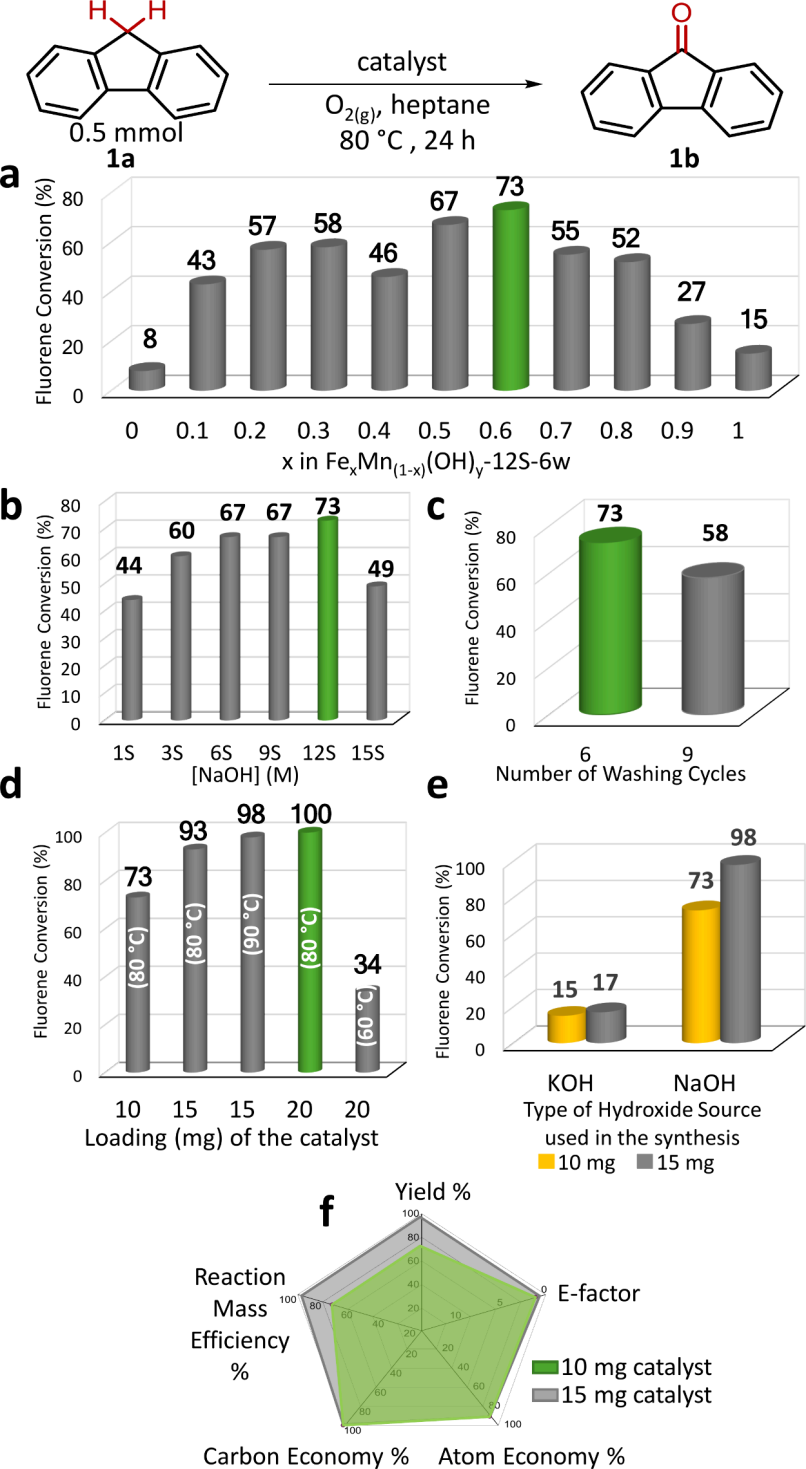
Catalytic performance results for the aerobic oxidation of fluorene (**1a**) to fluorenone (**1b**). (a) Fe_*x*_Mn_(1–*x*)_(OH)_*y*_-*12S-6w* (10 mg) catalysts prepared with different nominal Fe/Mn cation ratios; (b) Fe_0.6_Mn_0.4_(OH)_*y*_-*nS-6w* (10 mg) catalysts prepared with different NaOH(aq) concentrations; (c) Fe_0.6_Mn_0.4_(OH)_*y*_-*12S-nw* (10 mg) catalysts washed six or nine times. (d) Influence of catalyst loading and reaction temperature on catalytic activity (Fe_0.6_Mn_0.4_(OH)_*y*_-*12S-6w*). (e) Effect of the type of base (NaOH vs KOH) used in the synthesis on catalytic activity (Fe_0.6_Mn_0.4_(OH)_*y*_-*12S-6w*,10 and 15 mg, 24 h, 80 °C). (f) Green chemistry metrics for the optimized 10 and 15 mg Fe_0.6_Mn_0.4_(OH)_*y*_-*12S-6w* catalyst (24 h, 80 °C). The % conversion values were determined by ^1^H NMR spectroscopy (average values of two independent runs for each reaction are reported) after 24 h of reaction duration. (*S* represents a NaOH(aq) concentration of 2.06 M).

Figure 1 presents the results of catalytic performance tests for the aerobic oxidation of fluorene (**1a**) to fluorenone (**1b**) on monometallic and bimetallic hydroxide catalysts, carried out by varying the optimization parameters.

#### 3.1.1 Effect of the Nominal Fe/Mn Metal Cation Ratio

Figure 1a compares the impact of the Fe/Mn nominal metal cation ratio on the catalytic performance in the aerobic oxidation of 0.5 mmol of fluorene (**1a**) that was carried out using 10 mg of the Fe_*x*_Mn_(1–*x*)_(OH)_*y*_-*12S-6w* catalysts prepared with different metal loadings. It should be noted that the ^1^H NMR spectra recorded after each reaction indicated solely the presence of fluorene (**1a**) and its oxidation product fluorenone (**1b**) with no other detectable side products, meaning a very high selectivity for the tested reactions. Figure 1a revealed that two benchmark monometallic catalysts (i.e., Fe(OH)_*y*_ and Mn(OH)_*y*_) exhibited the lowest catalytic activities for fluorene (**1a**) oxidation with conversion values of 15 and 8%, respectively. The incorporation of only 10 atom % Fe active sites into the monometallic Mn(OH)_*y*_ catalyst (i.e., Fe_0.1_Mn_0.9_(OH)_*y*_-*12S-6w*) resulted in a remarkable 5-fold increase in catalytic activity. Conversely, the addition of 10 at. % Mn sites to the monometallic Fe(OH)_*y*_ catalyst (i.e., Fe_0.9_Mn_0.1_(OH)_*y*_-*12S-6w*) yielded an improvement of nearly 2-fold. In both instances, the transition from monometallic hydroxides to bimetallic structures brought about a significant enhancement in aerobic fluorene oxidation, indicating a synergistic effect between Fe and Mn active sites in the Fe_*x*_Mn_(1–*x*)_(OH)_*y*_-*12S-6w* catalyst. As evident from Figure 1a, varying the relative metal loadings enables precise fine-tuning of the catalyst performance, suggesting that the catalytic properties of these nanomaterials can be adjusted by controlling the relative nominal compositions of different metal cations. With the slight exception of the Fe_0.4_Mn_0.6_(OH)_*y*_ catalyst, the relative nominal metal loading ratio follows a volcano-type trend in terms of catalytic activity, indicating the presence of an optimum cation ratio. Accordingly, the Fe_0.6_Mn_0.4_(OH)_*y*_ sample exhibits the highest catalytic activity in aerobic fluorene oxidation. Notably, using only 10 mg of the Fe_0.6_Mn_0.4_(OH)_*y*_-*12S-6w* catalyst led to the formation of the oxidation product fluorenone (**1b**) with 73% conversion.

#### 3.1.2 Impact of the NaOH(aq) Concentration

Following the identification of the optimal Fe/Mn nominal cation ratio in the catalyst structure, we investigated the impact of the NaOH(aq) concentration used in the catalyst preparation on catalytic activity. As illustrated in Figure 1b, the lowest and the highest concentrations of NaOH(aq) exhibited limited catalytic activities, with conversion values of 44% and 49% for NaOH(aq) concentrations of *1S* and *15S*, respectively, and the catalytic performance reached its maximum value, with a conversion value of 73%, for the Fe_0.6_Mn_0.4_(OH)_*y*_-*12S-6w* catalyst.

#### 3.1.3 Influence of the Number of Washing Cycles

The best performing catalyst in the catalytic activity tests presented in Figures 1a,b (i.e., Fe_0.6_Mn_0.4_(OH)_*y*_-*12S*) was subjected to washing with 1, 3, 6, and 9 subsequent cycles of deionized water before the final drying stage and used in the catalytic aerobic oxidation of fluorene (Figure 1c). Fewer washing cycles (i.e., 1 and 3 times) led to the formation of salt deposits on the samples which entirely eradicated the catalytic performance, thus they are not shown in Figure 1c. As will be discussed in detail in the forthcoming sections, the number of washing cycles has a significant effect on the surface coverage of residual Na^+^ species on the catalyst surface. While excessive amounts of Na^+^ species residing on the catalyst surface after 1 and 3 washing cycles are quite detrimental for the catalytic performance, Figure 1c clearly indicates that extensive removal of Na^+^ residues on the surface of the catalyst also leads to a decrease in the catalytic activity for the nine-times washed sample, suggesting the presence of an optimum number of washing cycles of 6.

#### 3.1.4 Influence of Catalyst Amount and Reaction Temperature

Deployment of the optimized catalyst (Fe_0.6_Mn_0.4_(OH)_*y*_-*12S-6w*) in the catalytic aerobic oxidation of fluorene with various catalyst amounts of 10, 15, and 20 mg and different reaction temperatures at 60, 80, and 90 °C revealed that the maximum conversion of 100% could be obtained using 20 mg of catalyst at 80 °C (Figure 1d) after 24 h of reaction duration.

#### 3.1.5 Influence of the Nature of the Alkali Metal Hydroxide Used in the Synthesis

Upon realizing the notable influence of the residual alkali metal species on the catalyst surface after optimized synthesis and washing protocols (Figure 1a-d), we investigated the effect of the nature of the type of alkali metal by performing Fe_0.6_Mn_0.4_(OH)_*y*_-*12S-6w* synthesis with KOH(aq) rather than NaOH(aq) (Figure 1e). Catalytic performance tests executed with the K-containing catalyst clearly indicated a much lower fluorene oxidation activity than Na-containing sample suggesting the importance of the choice of the alkali metal hydroxide used in the synthetic protocol. The superior catalytic activity of Na-containing catalysts can be attributed to the incorporation of alkali metals into the lattice and/or on the catalyst surface. When the ionic radius of alkali metal cations is similar to that of transition metal cations in the lattice, these alkali metal cations are more likely to be incorporated not only within the bulk of the crystal lattice but also on the catalyst surface.^30^ This enhanced incorporation of Na^+^ species, compared to K^+^, tends to favor Na-containing catalysts. As a result, K-containing catalysts do not show enhanced catalytic performance, likely due to differing surface concentrations of K^+^ compared to Na^+^ under identical reagent concentrations and synthesis conditions.

#### 3.1.6 Green Chemistry Metrics

In an attempt to highlight the sustainability characteristics of the currently synthesized Fe_0.6_Mn_0.4_(OH)_*y*_-*12S-6w* catalyst, we calculated various Green Chemistry Metrics of this catalyst in fluorene oxidation reaction (Figure 1f, and Table S3–S5).^21^ Definitions and the calculations of these metrics are provided in the Supporting Information Section. Radar plot in Figure 1f illustrates that values of these metrics improve with increasing catalyst amount, and for 15 mg of catalyst, a yield of 98%, an E-factor of 0.47, an atom economy of 91%, a carbon economy of 100% and a reaction mass efficiency of 77% could be achieved covering favorably almost the entire area of the radar plot.

### 3.2 Substrate Scope and Mechanistic Studies

In order to check the effectiveness of the newly developed catalyst, we investigated the catalytic C–H oxidation of various alkylarene substrates (Table 1). In all of these reactions, the optimized Fe_0.6_Mn_0.4_(OH)_*y*_-*12S-6w* catalyst was used in the presence of 1 bar of O_2_(g) as the stoichiometric oxidant. As discussed above, the oxidation of fluorene (**1a**, 0.50 mmol) with the use of 10 mg of the catalyst at 80 °C gave fluorenone (**1b**) with 73% conversion and complete selectivity (Table 1, entry 1). Gratifyingly, execution of this transformation with the use of 15 mg of catalyst at 80 and 90 °C increased the conversion values to 93 and 98%, respectively (entries 2 and 3). Isolation of the product of the latter reaction by column chromatography afforded fluorenone (**1b**) in 98% yield (entry 3), which indicated that there is an excellent correlation between the measured conversion values and isolated product yields. Next, we turned our attention to the catalytic oxidation of xanthene (**2a**). Whereas the conversion to xanthone (**2b**) was moderate at 80 °C (53%, entry 4), the oxidation product **2b** was isolated in 92% yield after purification by column chromatography when the reaction was performed at 110 °C and with a 15 mg catalyst loading (entry 5). Under the same conditions, the benzylic oxidation of isochroman (**3a**) was not successful, and isocromanone (**3b**) was observed to be formed with only 6% conversion as determined by ^1^H NMR spectroscopy (entry 6). With these results in hand, we then focused on the catalytic C–H oxidation of diarylmethane derivatives. Due to the difficulties that we faced during the ^1^H NMR measurements for conversion determination, we purified the final oxidation products of these reactions by column chromatography and reported in Table 1 the isolated product yields. Pleasingly, the catalytic oxidation of diphenylmethane (**4a**, 0.25 mmol) with the use of 37 mg of the catalyst at 130 °C afforded benzophenone (**4b**) in 87% yield (entry 7). In order to examine the effect of having electron donating and withdrawing substituents on the benzene rings, we prepared diarylmethane derivatives **5a** and **6a**. Under the same conditions, the oxidation of **5a** with the electron donating -OMe group proceeded with a lower yield (59%, entry 8), whereas the presence of the electron withdrawing -NO_2_ group on substrate **6a** was observed to have a positive effect on the reaction affording oxidation product **6b** in 93% yield (entry 9). We should also note that at the end of all reactions discussed above only the indicated products and unreacted starting materials, if any, were observed without any side products. Overall, the results clearly demonstrate that the optimized Fe_0.6_Mn_0.4_(OH)_*y*_-*12S-6w* catalyst could be utilized in the aerobic oxidation of a variety of alkylarenes with high yields.

**Table 1:**
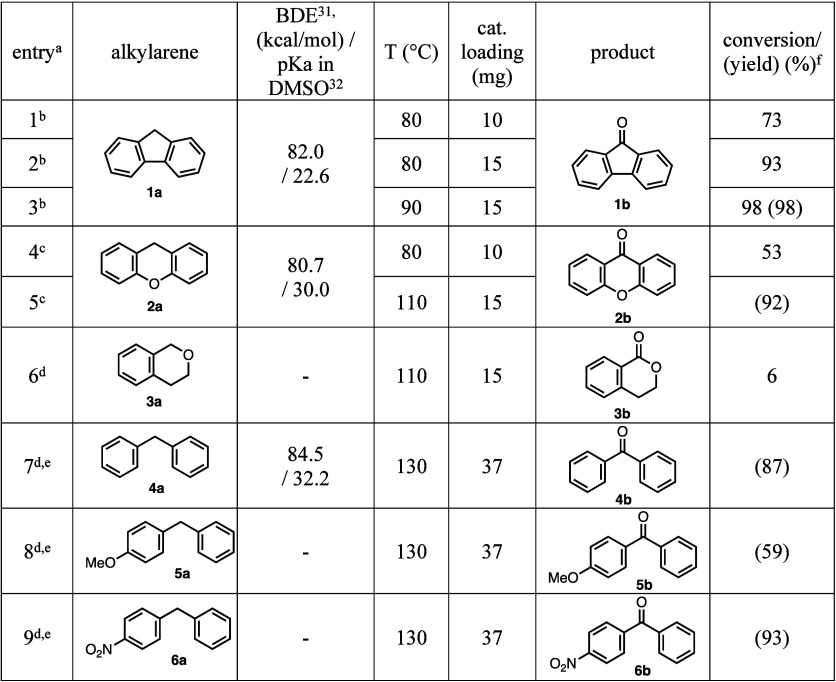
Aerobic Oxidation of Alkylarenes Catalyzed by the Fe_0.6_Mn_0.4_(OH)_*y*_-*12S-6w* Catalyst

a Reactions were carried out using 0.50 mmol of substrate and 2.0 mL of solvent under an atmosphere of *O*_2_(*g*) (1 bar) for 24 h.

b *n*-Heptane was used as the solvent.

c *n*-Octane was used as the solvent.

d Chlorobenzene was used as the solvent.

e 0.25 mmol of substrate was used in 1.0 mL of solvent.

f Conversion values were determined by ^1^H NMR spectroscopy, and isolated products were characterized using NMR spectroscopy (Figures S9–S17). Isolated product yields after purification by column chromatography are given in parentheses.

**Scheme 1:**
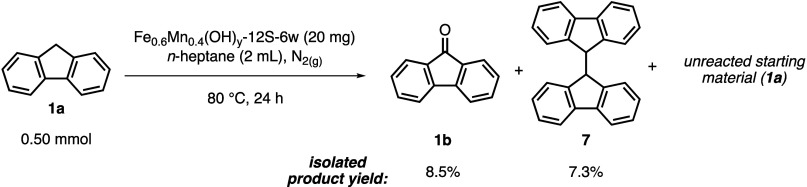
Effect of the Absence of O_2_(g) on the Reaction Outcome

To elucidate the mechanistic details of the Fe_0.6_Mn_0.4_(OH)_*y*_-*12S-6w*-catalyzed oxidation reactions, several control experiments were conducted. We previously demonstrated that the oxidation of fluorene (**1a)** did not proceed (<1% conversion) in the absence of a catalyst under the same reaction conditions, which justified that the presence of the catalyst is crucial for the activation of molecular oxygen and/or the reactant.^17^ In a subsequent series of control experiments, fluorene (**1a**, 0.50 mmol) oxidation was conducted under an inert atmosphere of N_2_(g) by using 20 mg of the Fe_0.6_Mn_0.4_(OH)_*y*_-*12S-6w* catalyst at 80 °C for 24 h. In order to make the solvent anhydrous, it was distilled from CaH_2_ under nitrogen. In addition, right before the experiment, the reaction mixture was deoxygenated by the application of the freeze–pump–thaw technique for five cycles to minimize molecular oxygen in the reaction medium. This experiment resulted in the formation of the ketone product **1b** and the dimerization product **7** in 8.5 and 7.3% isolated yields, respectively, whereas the majority of reactant fluorene (**1a**) was recovered intact (Scheme 1). These findings underscore the crucial role of molecular oxygen in the oxidation process. The limited ketone product formation might be attributed to incomplete solvent deoxygenation or the infiltration of adventitious oxygen into the reaction mixture.

**Scheme 2:**
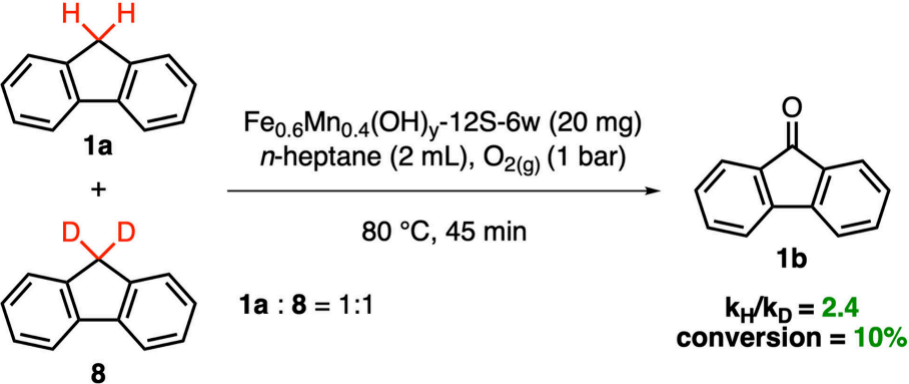
Kinetic Isotope Effect on Aerobic Fluorene Oxidation Using Fe_0.6_Mn_0.4_(OH)_*y*_-*12S-6w*

In an effort to probe the rate-determining step of the catalytic C–H oxidation reaction, a kinetic isotope effect experiment was conducted. Accordingly, a 1:1 mixture of fluorene **(1a)** and fluorene-*d*_*2*_**(8)** was subjected to the optimized reaction conditions using 20 mg of the Fe_0.6_Mn_0.4_(OH)_*y*_-*12S-6w* catalyst (Scheme 2). This created a reaction medium in which C–H and C–D bond functionalization occurred under identical conditions, ensuring more reliable results. The reaction was quenched after 45 min at approximately 10% conversion to stay in the kinetic region. The % consumption values of fluorene (**2a**) and fluorene-*d*_*2*_ (**4**) were determined through careful ^1^H NMR spectroscopic analysis. The value of the ratio of the nondeuterated to deuterated reaction rate constants (i.e., *k*_H_*/k*_D_) was found to be 2.4. This relatively low *k*_H_*/k*_D_ can still be considered as primary kinetic isotope effect (1° KIE or PKIE) in the reaction; as it is known that an extremely asymmetric linear transition state or a nonlinear transition state can significantly diminish the magnitude of the *k*_H_*/k*_D_ in PKIE.^33,34^ Particularly, when the C–H cleavage involves a nonlinear transition state, contributions from C–H bending modes with smaller force constants (and thus smaller vibrational frequencies) as compared to that of C–H stretching modes, can readily attenuate the magnitude of the observed *k*_H_*/k*_D_ in PKIE. Thus, it can be concluded that in the current work, the rate/product-determining step of the fluorine oxidation on Fe_0.6_Mn_0.4_(OH)_*y*_-*12S-6w* presumably involves the breaking of a C–H bond.^29,35−37^

**Scheme 3:**
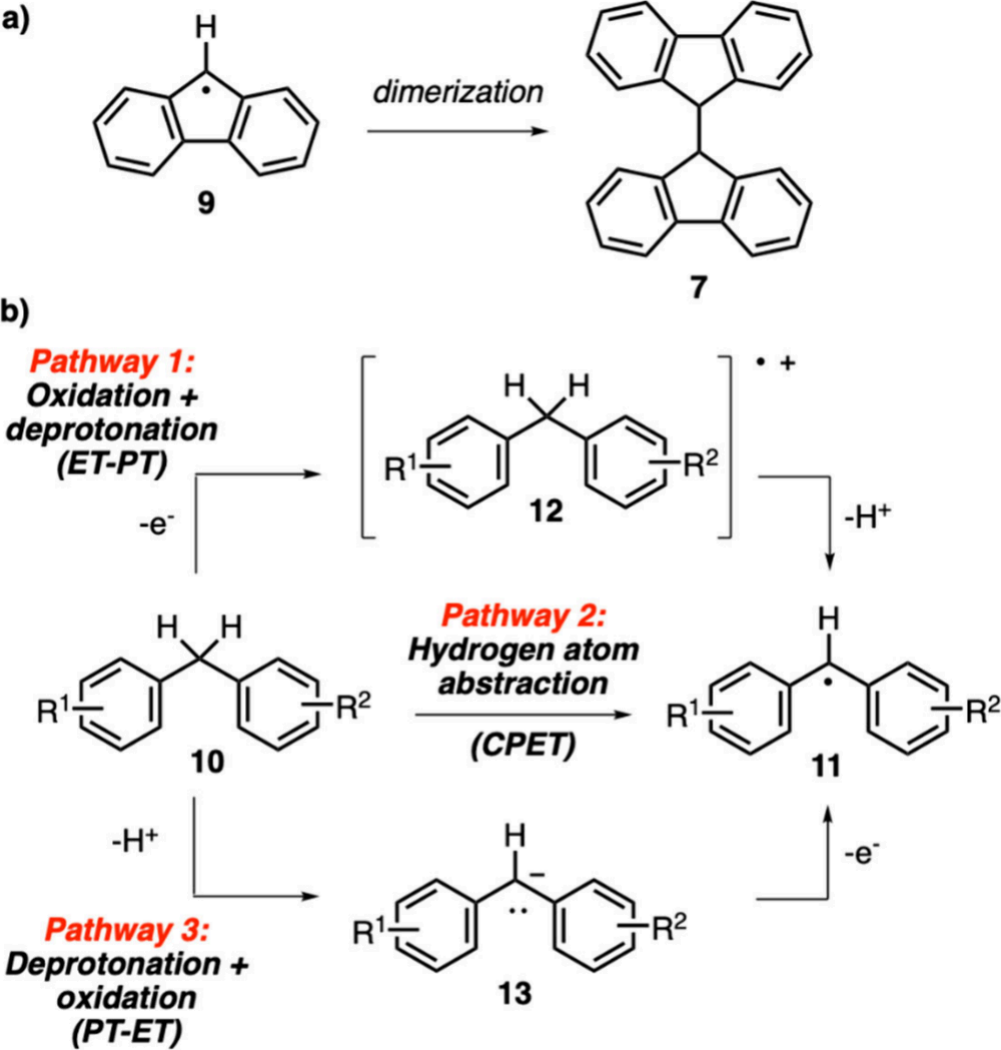
(a) The Structure of Radical Intermediate **9** Leading to the Dimerization Product **7**; (b) Possible Mechanistic Pathways for the Formation of Intermediate **11** from Alkylarene **10**

The experimental results mentioned above allowed us to evaluate the various mechanistic possibilities for the C–H activation process. The isolation of compound **7** in the control experiment shown in Scheme 1 points unequivocally to the formation of radical **9** as an intermediate during this reaction (Scheme 3a). Therefore, this observation supports the formation of radical intermediate **11** when alkylarene **10** is subjected to the catalytic oxidation conditions developed in this study (Scheme 3b). Based on earlier analyses of C–H activation reactions,^35,38^ three distinct mechanistic pathways can be proposed for this transformation: (1) ET-PT (electron transfer-proton transfer) pathway, which involves sequential oxidation and deprotonation processes; (2) CPET (coupled proton electron transfer) pathway, which involves a concerted hydrogen atom transfer process; and (3) PT-ET (proton transfer-electron transfer) pathway, which involves sequential deprotonation and oxidation processes. The ET-PT pathway (pathway 1) is expected to be favored with alkylarenes having electron-rich aryl groups, as they are easier to become oxidized. In this respect, the catalytic oxidation of xanthene (**2a**) was shown to proceed with a lower yield than fluorene (**1a**) despite the fact that the aryl groups of xanthene are more electron rich than those of fluorene (Table 1, entries 1 and 4). Likewise, among substrates **4a**, **5a**, and **6a**, compound **5a** gave the lowest yield in the oxidation reaction even though the methoxybenzene group of **5a** makes it more electron rich than the aryl groups of **4a** and **6a** (Table 1, entries 7–9). These considerations led us to rule out pathway 1 for the formation of radical **11**.

Next, we considered the CPET process (pathway 2) for the conversion of alkylarene **10** to radical **11** (Scheme 3b). Since this pathway involves a concerted hydrogen atom transfer, substrates with lower homolytic bond dissociation energies (BDE) are expected to be favored. However, even though xanthene (**2a**) has a lower BDE (80.7 kcal/mol) compared to fluorene (**1a**) (82.0 kcal/mol), it gave a lower yield in the catalytic oxidation reaction (Table 1).^31^ Moreover, both electron donating and withdrawing groups are known to stabilize radical intermediates.^39^ As a result, the reactions of substrates **5a** and **6a** were expected to proceed with yields higher than those of **4a** according to pathway 2 as their corresponding radical intermediates would be stabilized by the -OMe and -NO_2_ groups. However, in the catalytic oxidation reactions, substrate **6a** exhibited a higher reactivity than **4a**, whereas substrate **5a** with the electron donating -OMe group proceeded with a lower yield (Table 1). Overall, these observations do not lend support to pathway 2.

Finally, we focused on the PT-ET process (pathway 3) with an initial deprotonation step of alkylarene **10** followed by oxidation to give radical **11** (Scheme 3b). According to this pathway, more acidic substrates are expected to react more favorably in the oxidation reaction. Among the investigated substrates, fluorene (**1a**), xanthene (**2a**) and diphenylmethane (**4a**) were reported to have p*K*_a_ values of 22.6, 30.0, and 32.2 in DMSO, respectively; Table 1).^32^ The catalytic activities of these substrates displayed a good correlation with this acidity ranking, with fluorene being the most reactive substrate and diphenylmethane being the least reactive (Table 1). Moreover, among the diarylmethane derivatives **4a**, **5a**, and **6a**, substrate **6a** with the electron withdrawing -NO_2_ group is expected to be the most acidic, which also appeared to give the highest reaction yield (93%).

Based on the above discussions and the result of our KIE experiment, we propose that the catalytic oxidation method developed in this study proceeds through the PT-ET pathway (pathway 3, Scheme 3b) which involves an initial rate-determining deprotonation of alkylarene substrate **10** followed by oxidation to give radical intermediate **11**. A superoxide anion (O_2_^–^) which may be generated by the interaction of molecular oxygen with the metal catalyst,^16^ or hydroxide (OH^–^) anion from the catalyst may be acting as the base for the initial deprotonation step. In an elegant study reported by Borovik and co-workers in 2009, C–H bond cleavage reactions by two Mn-oxo complexes, namely [Mn^III^H_3_buea(O)]^2–^ and [Mn^IV^H_3_buea(O)]^−^, were investigated.^40^ Based on detailed mechanistic studies, the first complex with a KIE value of 2.6 was proposed to operate via a PT-ET mechanism, whereas the latter complex (KIE = 6.8) was proposed to follow a concerted CPET mechanism. Please note that, the mechanistic details in our previous catalytic C–H oxidation reaction with a NiMn-based LDH catalyst supported a concerted hydrogen-atom abstraction (CPET) mechanism with a KIE value of 5.7.^17^ As a result, our mechanistic conclusions for the current work along with our previous report,^17^ are in agreement with the analyses of Borovik and co-workers.^40^

### 3.3 Choice of Oxidant

Focusing on the choice of oxidant, even though molecular oxygen is our preferred oxidant, alternatives such as aerial oxygen or hydrogen peroxide can be considered. However, using air complicates data comparison due to its lower O_2_ partial pressure. Running the reaction in an open flask under air at 80–130 °C risks solvent evaporation, altering the reaction volume, despite using a condenser. Control experiments without O_2_ showed negligible catalytic conversion, underscoring its necessity. Hydrogen peroxide is also impractical due to its immiscibility with our solvents (heptane, octane, or chlorobenzene), creating a triphasic system. Organic peroxides like *tert*-butyl hydroperoxide (*t*-BuOOH) are soluble but pose significant safety risks at high temperatures. Therefore, molecular oxygen is more favorable, offering advantages in safety, cost, simplicity, and environmental compatibility, making it the optimal choice for our reaction system.

### 3.4 Structural Characterization of the Bimetallic Hydroxide Catalysts

**Figure 2:**
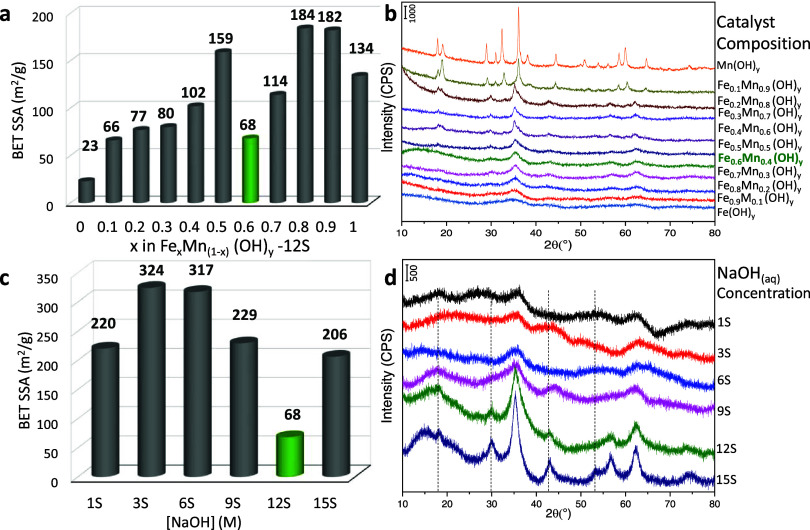
(a) SSA values and (b) XRD data for Fe_*x*_Mn_(1–*x*)_(OH)_*y*_-*12S-6w* catalysts. (c) SSA values and (d) XRD data for Fe_0.6_Mn_0.4_(OH)_*y*_-*nS-6w* catalysts synthesized with various NaOH(aq) concentrations.

Figure 2a depicts the SSA, while Figure 2b elucidates the influence of the nominal Fe/Mn cation ratio on the crystal structure of the Fe_*x*_Mn_(1–*x*)_(OH)_*y*_-*12S-6w* samples monitored via XRD. It is apparent that the monometallic Mn(OH)_*y*_ structure exhibits a well-ordered crystal structure, resulting in the lowest SSA of 23 m^2^/g. Conversely, the benchmark monometallic Fe(OH)_*y*_ catalyst exhibits a comparatively larger SSA (134 m^2^/g) with an amorphous structure. Additional XRD data given in Figure S2 regarding the benchmark monometallic Mn(OH)_*y*_ and Fe(OH)_*y*_ samples indicate the presence of Mn(OH)_2_ and Mn_3_O_4_ bulk phases in the Mn(OH)_*y*_ sample and the presence of Fe(OH)_3_ and FeOOH bulk phases in the Fe(OH)_*y*_ sample. A correlation between increasing manganese loading and increasing crystallinity can be readily observed in Figure 2b. A relevant trend is also generally valid in the SSA measurements, wherein an increase in manganese loading results in a decrease in SSA. It should be noted that iron oxide/hydroxide structures may display SSA values within 14–304 m^2^/g depending on the specific synthetic route employed.^41,42^ In contrast, manganese oxide/hydroxide structures typically exhibit SSA values ranging from 17–150 m^2^/g.^43−45^ For binary Fe–Mn oxide structures, reported SSA values often vary within the range of 1–90 m^2^/g.^46−48^ Interestingly, in the current work (Figure 2a), the most active catalyst (i.e., Fe_0.6_Mn_0.4_(OH)_*y*_-*12S-6w*), exhibits a relatively low SSA of 68 m^2^/g pointing out unique structural characteristics for this catalyst which are discussed in the forthcoming sections.

SSA and XRD data for Fe_0.6_Mn_0.4_(OH)_*y*_-*nS-6w* samples synthesized by using different NaOH(aq) concentrations are depicted in Figure 2c and Figure 2d, respectively. The lowest catalytic conversion observed for *1S* (Figure 1b) could be attributed to the inadequate concentration of NaOH(aq) that is less than that of the required amount for the formation of a metal hydroxide lattice (note that Fe and Mn aqueous precursor solutions are intrinsically acidic, and before the construction of the metal hydroxide lattice, acidic medium needs to be neutralized by the addition of a sufficient amount of NaOH). For the *9S* and *12S* cases, the concentration of NaOH(aq) used in the synthesis proved sufficient to construct a disordered iron–manganese hydroxide lattice (Figure 2d). Yet, for the best performing Fe_0.6_Mn_0.4_(OH)_*y*_-*12S-6w* catalyst, the presence of broad and convoluted diffraction features indicates the possible presence of additional metal oxide/metal hydroxide phases in the bulk structure of this catalyst. In the case of *15S*, extremely high NaOH(aq) led to the formation of additional diffraction signals at 17, 30, 42, and 52°, signifying the formation of a new inactive phase characterized by a high SSA.

**Figure 3:**
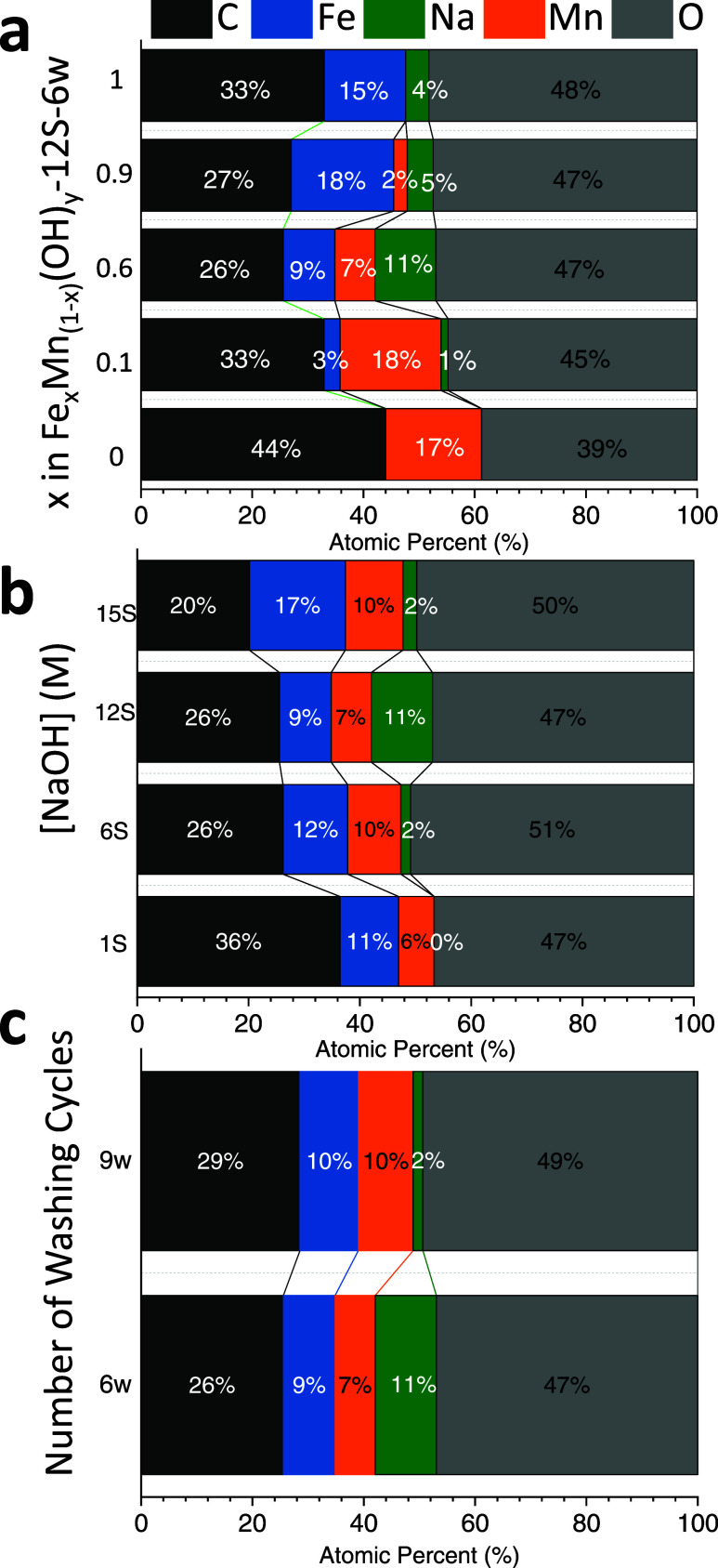
Surface atomic composition (at.%) values for a) Fe_*x*_Mn_1–*x*_(OH)_*y*_-*12S-6w* catalysts with various Fe and Mn loadings. b) Fe_0.6_Mn_0.4_(OH)_*y*_-*nS-6w* catalysts synthesized with various NaOH(aq) concentrations. (c) Fe_0.6_Mn_0.4_(OH)_*y*_-*12S-nw* catalysts synthesized with different number of washing cycles. (*S* represents a NaOH(aq) concentration of 2.06 M).

In an attempt to investigate the surface chemistry, electronic structure, and surface atomic composition of the FeMn-based hydroxide catalysts, XPS studies were conducted. Figure 3 illustrates the *surface* atomic compositions of the catalysts prepared for the optimization series obtained via XPS measurements. Figure 3a reveals an anticipated monotonically increasing surface concentration of Mn with an increasing nominal Mn precursor concentration used in the synthesis, along with a corresponding decrease in the Fe surface concentration. It is also worth mentioning that the relative *surface* atomic percentages of Fe and Mn presented in Figure 3a are in very good agreement with the nominal relative metal amounts used in the synthesis (Table S1 and Figure S3), which is in line with the argument that the surface of the catalyst is likely to be composed mostly of a bimetallic hydroxide structure. Figure 3b shows that varying NaOH(aq) concentrations used in synthesis has a visible influence on the surface Fe/Mn cation ratio. While the nominal Fe/Mn cation ratio is expected to be 1.5 for Fe_0.6_Mn_0.4_(OH)_*y*_, for extremely high (*15S*) or extremely low (*1S*) NaOH(aq) concentrations, the Fe/Mn ratio exceeds this value and becomes 1.7 or 1.8, respectively. On the other hand, for relatively moderate NaOH(aq) concentrations of *6S* and *12S*, where the catalytic activity converges and reaches its maximum value (Figure 1b), Fe/Mn ratio decreases below that of the nominal Fe/Mn ratio and approaches to a value of 1.2–1.3. Thus, the optimized Fe_0.6_Mn_0.4_(OH)_*y*_ -*12S-6w* catalyst surface is enriched with Mn as compared to the corresponding nominal Fe/Mn ratio used in the synthesis. Hence, it is clear that the NaOH(aq) concentration used in the synthesis protocol offers an additional synthetic degree of freedom to fine-tune the surface cation ratio on the catalyst surface with a direct impact on catalytic performance.

Figure 3c reveals the influence of washing cycles on the surface atomic compositions. It is apparent that extensive washing (i.e., *9w*) decreases the Fe/Mn ratio to 1.0, suggesting a relative loss of Fe or excessive enhancement of Mn on the catalyst surface. Notably, increasing the number of washing cycles significantly reduces the surface sodium ions. Remarkably, the best catalyst (i.e., Fe_0.6_Mn_0.4_(OH)_*y*_-*12S-6w*) exhibits a noticeably greater amount of sodium ions on its surface compared to other catalyst samples.

It is worth mentioning that we also investigated the *bulk* elemental composition of the Fe_*x*_Mn_1–*x*_(OH)_*y*_*-12S-6w* catalysts via ICP-MS (Figure S4). As expected from the rather disordered and poorly defined bulk structure of these materials evident by the weak and broad XRD signals presented in Figures 2b and 2d, the Fe/Mn bulk cation ratio measured via ICP-MS diverges more significantly from the corresponding nominal Fe/Mn ratios used in the synthesis as compared to that of the surface Fe/Mn ratios (Figure 3 and Figure S3) suggesting the possible presence of various additional metal oxide or metal hydroxide phases in the *bulk* catalyst structure.

**Figure 4:**
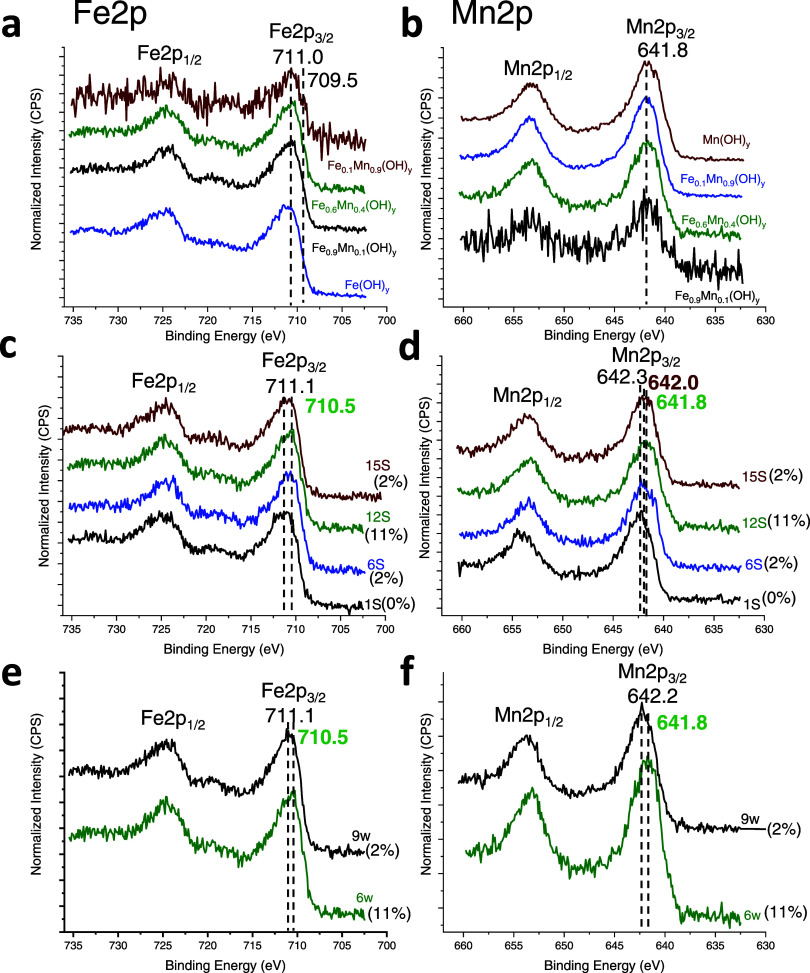
XPS data of Fe and Mn 2p for (a-b) Fe_*x*_Mn_(1–*x*)_(OH)_*y*_-*12S-6w* catalysts prepared with different nominal Fe/Mn cation ratios via the chemical precipitation; (c-d) Fe_0.6_Mn_0.4_(OH)_*y*_-*nS-6w* catalysts prepared with different NaOH(aq) concentrations, (e-f) Fe_0.6_Mn_0.4_(OH)_*y*_-*12S-nw* catalysts washed six and nine times. Numbers in parentheses in the plots represent the surface Na at.% on the catalysts.

Fe 2p and Mn 2p XPS spectra of the catalysts used in the catalytic performance optimization series are presented in Figures 4a-f. Biesinger and co-workers have previously discussed the complex characteristics of both Fe 2p and Mn 2p XPS spectra in the literature.^49^ This prior work emphasized the challenges inherent in X-ray photoelectron spectroscopic analysis of the first-row transition metal oxides/hydroxides, particularly that of Fe, owing to factors such as (i) complex multiplet splitting, (ii) peak asymmetries in 2p_3/2_ and 2p_1/2_ features, (iii) overlapping/uncertain binding energies, (iv) need for complex Shirley background offsets and fitting parameters, and (v) presence of additional satellites and plasmon loss structures. Given these complexities,^49−53^ we will provide overall assignments regarding the Fe 2p and Mn 2p XPS data. Specifically, the B.E. of the Fe 2p_3/2_ signal has been established in the energy range of 706.5–707.0 eV for metallic iron (Fe^0^), 709.5–710.3 eV for Fe^2+^, and 710.6–711.4 eV for Fe^3+^. Additionally, a broad shakeup satellite centered at 719.8 eV is indicative of Fe^3+^, while a satellite at *ca*. 715 eV is characteristic of Fe^2+^ species. Figure 4a shows the Fe 2p spectra belonging to the Fe_*x*_Mn_1–*x*_(OH)_*y*_*-12S-6w* catalysts with selected Fe/Mn nominal cation ratios. The presence of Fe is characterized by distinctive Fe 2p_3/2_ peaks around 711.0–711.5 eV, serving as an indication of predominantly Fe^3+^ species. Notably, the existence of a lower B.E. shoulder around 709.5 eV suggests the coexistence of Fe^2+^ minority species on the catalyst surface.

Effect of the NaOH(aq) concentration used in the synthesis on the Fe 2p B.E. values of the Fe_0.6_Mn_0.4_(OH)_*y*_-*nS-6w* catalysts are depicted in Figure 4c. While the maxima of the Fe 2p_3/2_ XP signals are observed at 711.1 eV for *1S* and *15S* samples, Fe 2p_3/2_ B.E. values shift to a lower value of *ca*. 710.5 eV for the *6S* and *12S* catalysts. This observation is in line with the corresponding catalytic activity trends given in Figure 1b revealing a higher catalytic performance for *6S* and *12S* catalysts where surface Fe^3+^ sites are richer in electron density as compared to those of the less active catalysts.

Figure 4e illustrates the impact of the number of washing steps on the corresponding Fe 2p XP spectra where the surface atomic Na at.% values are also provided in parentheses. Notably, the best catalyst (i.e., Fe_0.6_Mn_0.4_(OH)_*y*_-*12S-6w*) exhibits a significant surface concentration of Na (11 at. %), leading to a Fe 2p_3/2_ signal at 710.5 eV which is −0.6 eV lower than that of Fe_0.6_Mn_0.4_(OH)_*y*_-*12S-9w* catalyst with a Na content of 2 at. %, whose Fe 2p_3/2_ signal B.E. appears at a higher value of 711.1 eV. This observation is consistent with the electronic promotion effect of Na^+^ species which lead to electron transfer from Na to Fe surface cations resulting in the formation of Fe-rich cationic surface sites and boost catalytic performance (Figure 1c).

The literature suggests that a comprehensive analysis of the Mn oxidation state should consider three parameters: (i) the binding energy of the 2p_3/2_ peak, which increases progressively with the oxidation state of Mn, (ii) the position of the 2p_3/2_ satellite structure, and (iii) the Mn 3s multiplet splitting value.^54−57^ Hastuti et al. reported that, in Fe-doped MnO_2_, Mn 2p_3/2_ signals at 640 eV indicate the presence of Mn^2+^ species, while Mn^3+^ species were observed at 1–2 eV higher binding energies.^58^ Structures containing Mn^4+^ exhibited characteristic Mn 2p_3/2_ signals at 644 eV. Figure 4b shows that the Mn 2p_3/2_ signals of the selected Fe_*x*_Mn_(1–*x*)_(OH)_*y*_-*12S-6w* catalysts yield a similar B.E. value of 641.8 eV suggesting the predominant presence of Mn^3+^ species with an asymmetry toward lower B.E. values indicating the presence of Mn^2+^ minority species. Figure 4d illustrates the influence of NaOH(aq) used in the synthesis of Fe_0.6_Mn_0.4_(OH)_*y*_-*nS-6w* catalysts on Mn 2p_3/2_ B.E. values. It is apparent that the Mn 2p_3/2_ B.E. values for the *1S, 6S*, and *12S*, samples decrease in a monotonic manner from 642.3 to 642.0 eV and to 641.8 eV, respectively, while Mn 2p_3/2_ signal for the *15S* appears at 642.0 eV. Comparing these Mn 2p_3/2_ B.E. values in Figure 4d with the corresponding catalytic performance results in Figure 1b clearly suggests a similar trend in catalytic conversion values indicating that the catalytic performance maximizes at the lowest Mn 2p_3/2_ B.E. of 641.8 eV observed for Fe_0.6_Mn_0.4_(OH)_*y*_-*12S-6w*. Figure 4f depicts the influence of the washing cycles in the electronic structure of the surface Mn species where extensive washing (*i.e., 9w*) and loss of surface Na species lead to an increase in Mn 2p B.E. values. Overall analysis of the Fe 2p and Mn 2p XPS data suggests that the catalytic performance is typically optimized in the prominent presence of electron enriched Fe^3+^ and Mn^3+^ species on the Fe_0.6_Mn_0.4_(OH)_*y*_-*12S-6w* surface which are generated by the electron transfer from Na species abundant on this catalyst.

**Figure 5:**
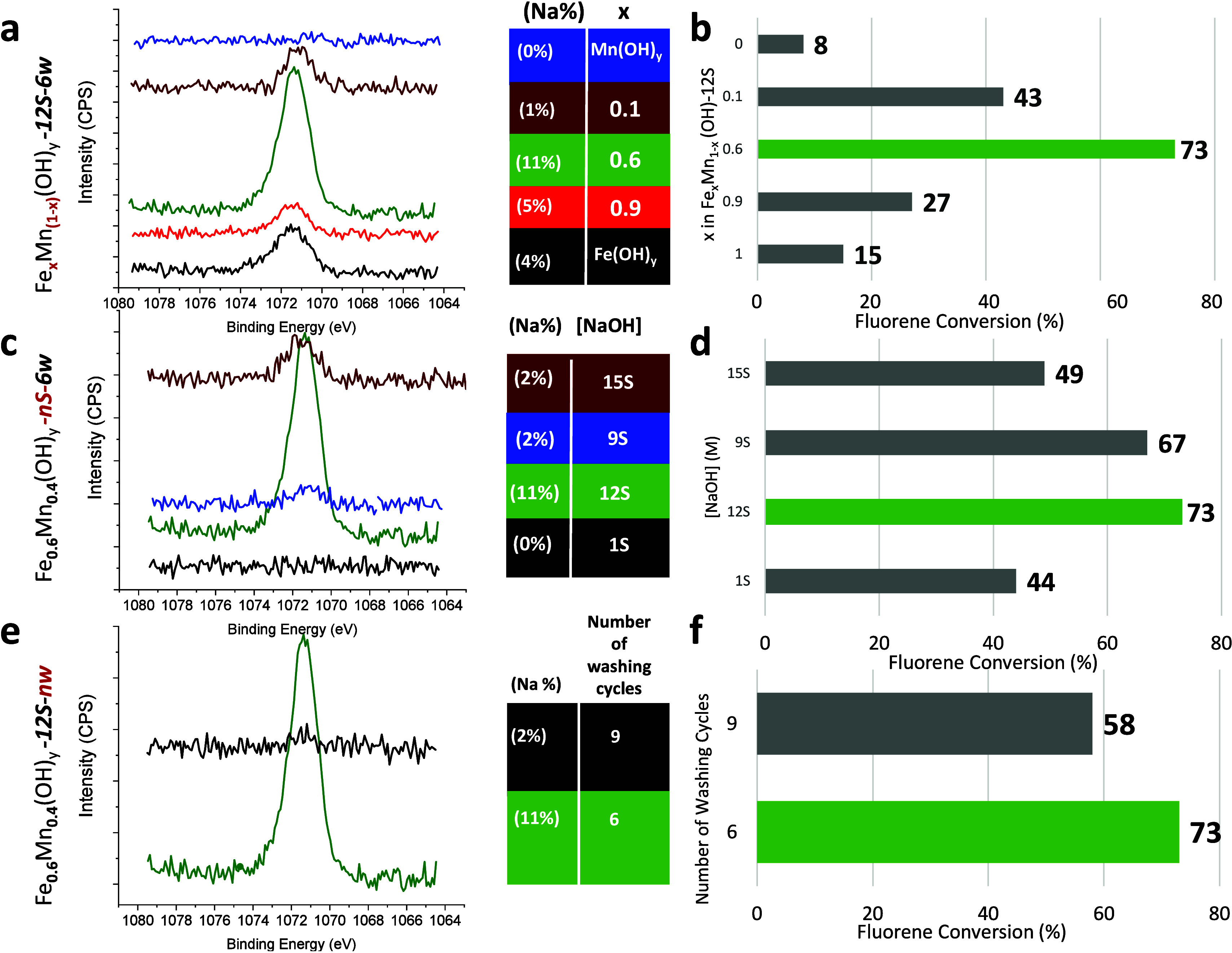
(a) Na 1s XPS data and (b) corresponding catalytic activity data for Fe_*x*_Mn_(1-x)_(OH)_*y*_-*12S-6w* catalysts in the aerobic oxidation of fluorene (**1a**) to fluorenone (**1b**) prepared with different nominal Fe/Mn cation ratios. (c) Na 1s XPS data and (d) corresponding catalytic activity data for Fe_0.6_Mn_0.4_(OH)_*y*_-*nS-6w* catalysts prepared with different NaOH(aq) concentrations. (e) Na 1s XPS data and (f) corresponding catalytic activity data for Fe_0.6_Mn_0.4_(OH)_*y*_-*12S-nw* catalysts washed six or nine times.

After having observed a significant presence of sodium ions on the surface of the optimal catalyst, our focus shifted to exploring the influence of Na residues on the catalytic activity. Figure 5 illustrates the Na 1s XPS spectra for the selected catalysts along with the catalytic activities observed in the optimization series. A thorough analysis of the XPS data indicates that the surface Na concentration exerts a significant effect on the catalytic activity through electron promotion to the metals, as further supported by the red shift in the corresponding Mn 2p and Fe 2p XP spectra presented in Figure 4. This enhanced catalytic activity is observed particularly in optimal catalyst Fe_0.6_Mn_0.4_(OH)_*y*_-*12S-6w* with the highest Na surface concentration. The introduction of sodium has been shown to alter the surface work function and strengthen the interaction between molecular oxygen and the surface metal. This may result in an increase in the adsorption energy of O_2_, subsequently lowering the energy barrier of the transition states for the oxidation process.^47^ Furthermore, the addition of Na to the catalyst surface might weaken the lattice metal–oxygen bond strength and promote the mobility and availability of lattice oxygen species contributing to the catalytic activity.^18^ Additionally, sodium can increase the electron density of the catalyst surface, facilitating the electrophilic adsorption of reactants. A significant amount of Na^+^ presence on the surface may also explain the unusually low SSA of the best performing Fe_0.6_Mn_0.4_(OH)_*y*_-*12S-6w* catalyst (Figures 2a and 2c) where Na^+^ species can block some of the pores and decrease the SSA. These multifaceted effects of sodium on the catalyst surface are likely to contribute to the observed improvements in catalytic performance. We have recently shown that the use of solely NaOH (in the absence of any other catalyst) in the aerobic oxidation of fluorene (**1a**) did not result in any product formation under the same reaction conditions that are employed in the current work (i.e., at 80 °C and heptane as solvent).^17^ In light of these control experiments, it is apparent that Na^+^ species are likely to function as an electronic promoter rather than a catalytic active site in the aerobic oxidation of fluorene (**1a**).

**Figure 6:**
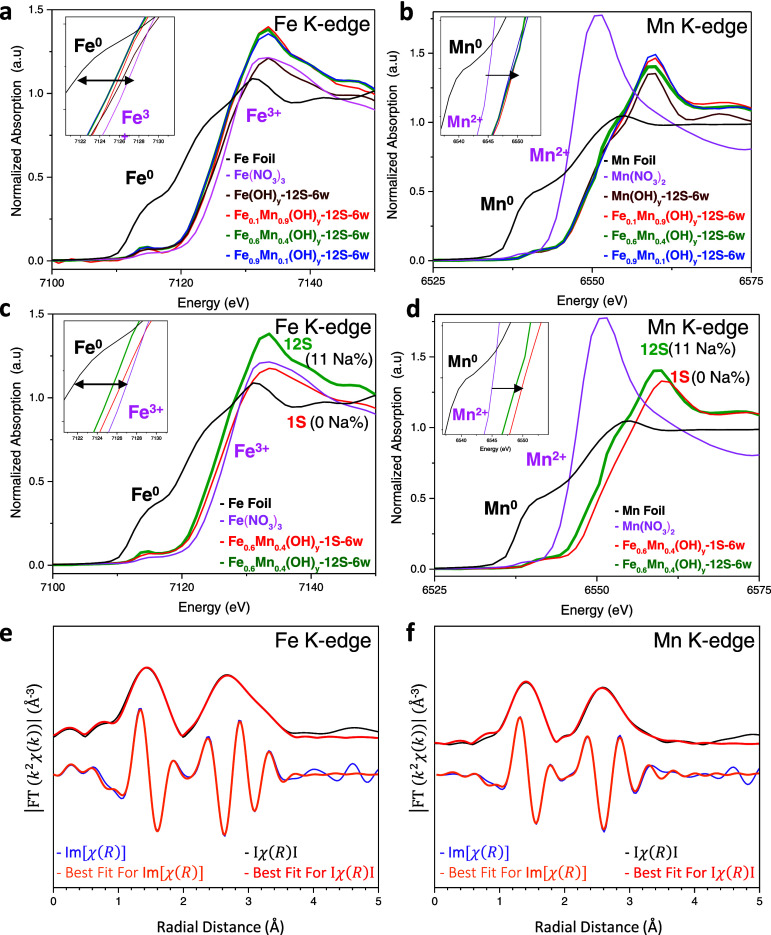
K-edge XANES spectra of Fe (a,c) and Mn (b,d) for (a,b) Fe_*x*_Mn_(1–*x*)_(OH)_*y*_-*12S-6w* catalysts prepared with different (e.g., *x* = 0.1, 0.6, 0.9) nominal Fe/Mn cation ratios; (c,d) Fe_0.6_Mn_0.4_(OH)_*y*_-*nS-6w* catalysts prepared with different NaOH(aq) concentrations. EXAFS results for the Fe_0.6_Mn_0.4_(OH)_*y*_-*12S-6w* catalyst at the Fe (e) and Mn (f) K-edges, showing the best fit in the form of Fourier-transform (FT) of the *k*^2^χ(*k*) and imaginary part (Im[χ(*R*)]) of the χ(*R*) spectra.

XANES measurements provided valuable insights into the *bulk* electronic structure of the synthesized catalysts. In X-ray absorption spectroscopy, the pre-edge region (*ca*. 7115 eV) is commonly utilized to elucidate 1s → 3d transitions and the K-edge (main line) corresponds to the 1s → 4s transitions.^59,60^ The inflection points of the main line are referred to as the edge energy. The K-edges exhibit evidence of changes in coordination environment.^61−63^ The negative shift in the edge location is interpreted as a decrease in the average oxidation state. Figures 6a,b illustrates the XANES spectra for Fe and K-edges for various Fe_*x*_Mn_1–*x*_(OH)_*y*_*-12S-6w* catalysts. It can be seen in Figures 6a,b that all samples exhibit an oxidation state of Fe between +2 and +3 and an Mn oxidation state greater than +2. For the optimized Fe_0.6_Mn_0.4_(OH)_*y*_-*12S-6w* catalyst, the calculated average bulk oxidation state is +2.1 for Fe and +2.4 for Mn, based on the linear combination fitting of the absorption edge positions in the XANES curves (Figures S5a,b). These findings are in general accordance with the current XPS data (Figure 4) indicating the coexistence of both divalent and trivalent Fe and Mn surface species. Figures 6c,d shows the influence of the amount of NaOH used during synthesis. As observed in X-ray photoelectron spectroscopy (XPS), an increase in the quantity of NaOH leads to a red shift in both Fe and Mn edges, signifying a decrease in their respective *bulk* oxidation states, presumably due to the incorporation of Na^+^ species into the bulk lattice. While the bulk and surface oxidation states of Fe and Mn species of the catalysts determined by XANES (Figure 6) and XPS (Figures 4,5) somewhat differ, the observed trends and shifts in oxidation states due to the presence of Na species are similar. Thus, current XANES and XPS data indicate that the substantial presence of Na induces a decrease in both the *bulk* and *surface* oxidation states of Fe and Mn species due to the electron transfer from Na to the Fe and Mn species.

Figures 6e,f present the *k*^2^-weighted χ(k) EXAFS spectra at the Fe and Mn K-edges for the Fe_0.6_Mn_0.4_(OH)_*y*_-*nS-6w* catalyst, respectively. The EXAFS data and fitted curves, which were not corrected for the scattering phase shift (∼0.5 Å), provide valuable insights into the local atomic structure. The structural parameters obtained from EXAFS fitting are summarized in Tables S6 and S7. The fitting results indicate that the first coordination shell, located near 2 Å in both the Fe and Mn K-edge spectra, reveals M–O (M = Fe, Mn) interatomic distances. These distances correspond to M–O scatterings, primarily associated with tetrahedral sites, with Fe–O and Mn–O bond lengths of 1.96 and 1.92 Å, respectively.^64−66^ Additionally, contributions from hydrogen atoms at longer distances were observed, potentially related to Fe–OH or Mn–OH coordination, with Fe–H at 2.88 Å and Mn–H at 2.45 Å. The coordination numbers deviate slightly from those of the ideal *bulk* structure, suggesting local disorder around the Fe and Mn atoms, likely due to structural defects. The coordination number (CN) for Fe–O is 3.51, and for Mn–O, it is 2.7 (Tables S6 and S7). This local disorder implies that both Fe and Mn are situated in a more defective, less ordered sites.^67^ These EXAFS results are consistent with the amorphous structure suggested by XRD data as well as the oxidation states derived from XANES data. In the second shell fitting, it was not possible to distinguish between Fe and Mn contributions due to their similar atomic numbers, making it difficult to differentiate between the local environments of the absorber atoms.^68^

In order to demonstrate the thermal stability of the optimized Fe_0.6_Mn_0.4_(OH)_*y*_-*12S-6w* catalyst within the temperature window of interest, we carried out thermal gravimetric analysis (TGA) measurements (Figure S6). Analysis of the TGA data (see SI section) indicates that the optimized catalyst is thermally stable under the currently used reaction temperatures. Regarding pH stability, please note that the current study does not exploit any aqueous medium in the catalytic reactions where pH control could be relevant. In contrast, currently presented catalytic performance experiments involve organic (nonaqueous) media which often involve nonpolar solvents (e.g., heptane and octane) where pH stability is not relevant. At any rate, it is worth mentioning that the typical pH of the reaction conditions where heptane was used as the solvent was ca. 7.9, and the addition of the reactant (fluorene) changed the pH to 6.8 at room temperature and 6.5 at 80 °C. TEM images of the optimized Fe_0.6_Mn_0.4_(OH)_*y*_-12S-6w catalyst are shown in Figure S7. Analysis of these images revealed no distinct morphological features that can be directly correlated to the enhanced catalytic performance of the optimized catalyst.

In our quest to assess the reusability of our fine-tuned catalyst in fluorene oxidation reactions, we carried out experiments to investigate both the reusability and applicability of regeneration protocols. These experiments involved running catalytic reactions without regenerating the catalyst in the oxidation of fluorene (**1a**). Notably, while the first catalytic cycle yielded a high conversion rate (89%) to fluorenone (**1b**), the catalytic activity notably decreased in the second cycle, reaching only a 9% conversion (Figure S8). Through numerous trials, we found that heat-based procedures were ineffective in restoring the catalytic activity of the optimized catalyst. Consequently, we transitioned to a room temperature regeneration protocol, which yielded 45% conversion after regeneration (Figure S8).

## 4 Conclusion

In summary, we developed an effective catalytic method for the oxidation of benzylic C–H bonds utilizing Fe_0.6_Mn_0.4_(OH)_*y*_-*12S-6w*, a novel precious metal-free mixed metal hydroxide catalyst. The optimization of the catalyst was conducted systematically, considering the Fe/Mn metal cation ratio, the concentration of NaOH(aq) used for catalyst synthesis, and catalyst washing during the synthesis. The method relies on molecular oxygen as the sole stoichiometric oxidant and works successfully with a low catalyst loading in high yields for a variety of alkylarene substrates. Detailed mechanistic studies including a kinetic isotope effect (KIE) experiment as well as analysis of the reactivities of the investigated substrates support the involvement of a base-assisted PT-ET process that consists of an initial rate-determining deprotonation of the alkylarene substrate followed by oxidation to give a radical intermediate in the key C–H bond cleavage process. The optimized Fe_0.6_Mn_0.4_(OH)_*y*_-*12S-6w* catalyst was characterized by (i) Fe cationic sites containing a mixture of Fe^2+^ and Fe^3+^ species, where Fe^3+^ species are the surface and Fe^2+^ bulk dominating species, (ii) the presence of Mn^2+^ and Mn^3+^ species working synergistically with Fe^3+/2+^ cations, (iii) a relatively low specific surface area of 68 m^2^/g, (iv) a relatively disordered and defective crystal structure consisting of bimetallic hydroxides as well as additional oxide/oxyhydroxide phases, and (v) residual Na^+^ surface species enabling electronic promotion of the cationic active sites via electron donation. Ultimately, the present results clearly indicate that chemically fine-tuned mixed metal hydroxide systems are promising catalytic alternatives for low-temperature liquid-phase organic reactions.

## Supporting Information Available

ICP-MS, TGA, additional XPS, XRD, XANES, and EXAFS data for the characterization of catalysts; regeneration protocol and catalytic performance of regenerated catalyst; experimental procedures, characterization data and NMR images for the oxidation products (PDF)
