# Supplementary material for: Na-Promoted Bimetallic Hydroxide Nanoparticles for Aerobic C–H Activation: Catalyst Design Principles and Insights into Reaction Mechanism
Source: ACS Appl Mater Interfaces. 2024 Oct 25;16(44):60151–65. doi: 10.1021/acsami.4c11070 (PMC11551905; doi:10.1021/acsami.4c11070)
Supplement: Supplementary file 1 — am4c11070_si_001.pdf [file am4c11070_si_001.pdf]

Supporting Information

Na-Promoted Bimetallic Hydroxide Nanoparticles for Aerobic C-H Activation: Catalyst Design Principles and Insights into Reaction Mechanism

---

Beyzanur Erdivan,[‡] Eylul Calikyilmaz,[‡] Suay Bilgin,[‡] Ayse Dilay Erdali,[‡] Damla Nur Gul,[‡] Kerem Emre Ercan, [‡¶] Yunus Emre Türkmen,[*,‡,§] and Emrah Ozensoy[*,‡,§]

[‡] Bilkent University, Department of Chemistry, Faculty of Science, 06800, Ankara, Türkiye

[¶] Roketsan Inc., 06780 Elmadag, Ankara, Türkiye

[§] UNAM - National Nanotechnology Research Center and Institute of Materials Science and Nanotechnology, Bilkent University, 06800, Ankara, Türkiye

**Corresponding Authors:*

Emrah Ozensoy (email: ozensoy@fen.bilkent.edu.tr)

Yunus Emre Türkmen (email: yeturkmen@bilkent.edu.tr)

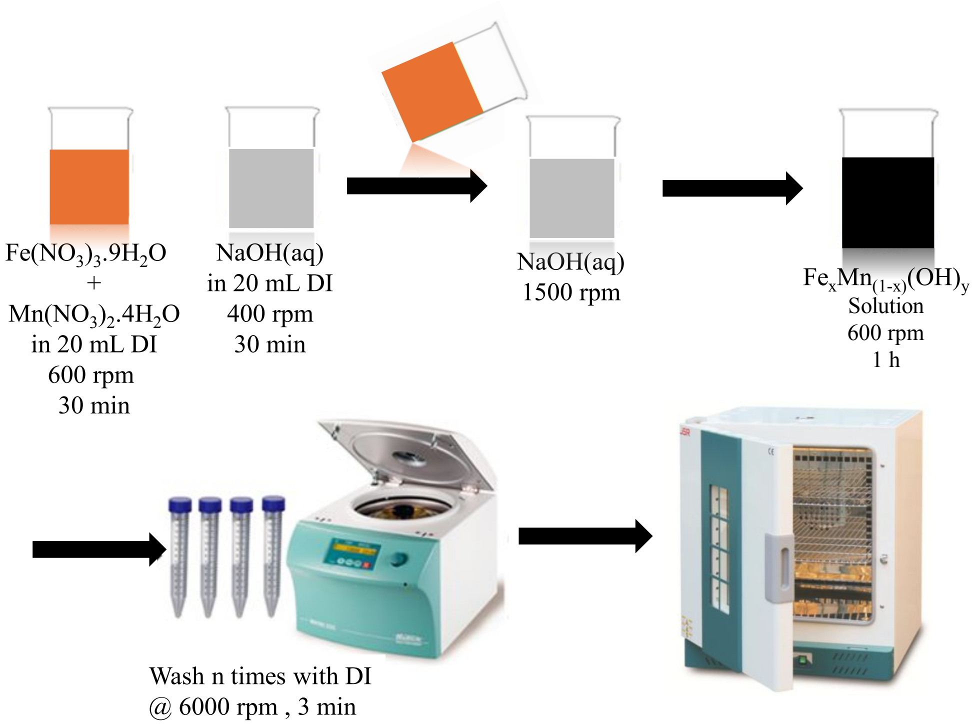

Figure S1. Schematic representation of $Fe_xMn_{(1-x)}(OH)_y$ synthesis via chemical coprecipitation method.

**Sample**	**Mass of $Fe(NO_3)_3.9H_2O$ (g)**	**Mass of $Mn(NO_3)_2.4H_2O$ (g)**	**Mass of NaOH (g)**
**$Mn(OH)_y$**	-	5.171	19.77
**$Fe_{0.1}Mn_{0.9}(OH)_y$**	0.6656	3.722	19.77
**$Fe_{0.2}Mn_{0.8}(OH)_y$**	1.331	3.308	19.77
**$Fe_{0.3}Mn_{0.7}(OH)_y$**	1.997	2.895	19.77
**$Fe_{0.4}Mn_{0.6}(OH)_y$**	2.662	2.481	19.77
**$Fe_{0.5}Mn_{0.5}(OH)_y$**	3.329	2.068	19.77
**$Fe_{0.6}Mn_{0.4}(OH)_y$**	3.995	1.655	19.77
**$Fe_{0.7}Mn_{0.3}(OH)_y$**	4.660	1.241	19.77
**$Fe_{0.8}Mn_{0.2}(OH)_y$**	5.326	0.827	19.77
**$Fe_{0.9}Mn_{0.1}(OH)_y$**	5.992	0.414	19.77
**$Fe(OH)_y$**	5.548	-	19.77

Table S1. The amounts of precursors used in the optimization of Fe/Mn cation ratio in the chemical coprecipitation synthesis method.

**Stoichiometry**	**Molarity (mol/L)**	**Amount (mol)**	**NaOH (g)**	**KOH (g)**
**1S**	2.06	0.0411	1.65	-
**3S**	6.18	0.1236	4.94	-
**6S**	12.36	0.2471	9.88	-
**9S**	18.53	0.3707	14.83	-
**12S**	24.72	0.4942	19.77	27.73
**15S**	30.89	0.6178	24.71	-

Table S2. The amounts of NaOH used in the optimization of NaOH concentration utilized in chemical coprecipitation synthesis method with different stoichiometric ratios.

Green Chemistry Metrics

The E-factor of a process represents the waste-to-product ratio, with an ideal E-factor value of 0 indicating minimal waste production. Atom economy indicates the proportion of reactants retained in the final product. Carbon economy measures the ratio of carbon in the useful product to the total carbon used in reactants. Reaction mass efficiency expresses the percentage of desired product mass compared to the total mass of all reactants used. Below are the calculation formulas for each metric. The calculations are based on the assumption of complete solvent recycling.

$$E - factor = \frac{mass\ of\ total\ waste}{mass\ of\ product}$$

$$Atom\ economy = \frac{molecular\ mass\ of\ desired\ product}{molecular\ masses\ of\ reactants}\ x\ 100\%$$

$$Carbon\ economy = \frac{number\ of\ carbon\ atoms\ in\ desired\ product}{number\ of\ carbon\ atoms\ in\ reactants}\ x\ 100\%$$

$$Reaction\ mass\ efficiency = \frac{actual\ mass\ of\ desired\ product}{mass\ of\ reactants}\ x\ 100\%$$

10 mg Catalyst Loading					
		**Molecular Weight (g/mol)**	**Amount (mmol)**	**Amount (g)**	**Number of carbon atoms**
**Reactant**	fluorene	166.223	0.50	0.083	13
**Oxidant**	oxygen	31.999	1.00 (1 bar)	0.032	0
**Solvent**	n-heptane	100.210	13.65 (2 ml)	1.368	7
**Recycled solvent**	n-heptane	-	-	1.368	-
**Product**	fluorenone	180.192	0.50	0.090	13
**Yield:**	73%		0.36	0.066	
**Catalyst**	$Fe_{0.6}Mn_{0.4}(OH)_y$-12S-6w	-	-		
**E-factor**	((83.1 + 31.9 + 10.0 + 1368.0) - (1368.0 + 65.8)) / 65.8 = 0.90				
**Atom economy**	(180.132 / (166.223 + 31.999)) x 100 = 91%				
**Carbon economy**	100%				
**Reaction mass efficiency**	65.77 / (83.11 + 31.9) x 100 = 57%				

Table S3. Green Chemistry metrics calculations for 10 mg loading of the $Fe_{0.6}Mn_{0.4}(OH)_y$-*12S-6w* catalyst.

15 mg Catalyst Loading					
		**Molecular Weight (g/mol)**	**Amount (mmol)**	**Amount (g)**	**Number of carbon atoms**
**Reactant**	fluorene	166.223	0.5	0.083	13
**Oxidant**	oxygen	31.999	1.00 (1 bar)	0.032	0
**Solvent**	n-heptane	100.210	13.65 (2 mL)	1.368	7
**Recycled solvent**	n-heptane	-	-	1.368	-
**Product**	fluorenone	180.192	0.50	0.090	13
**Yield:**	98%		0.49	0.088	
**Catalyst**	$Fe_{0.6}Mn_{0.4}(OH)_y$-12S-6w	-	-		
**E-factor**	((83.1 + 31.9 + 15.0 +1368.0) - (1368.0 + 88.3)) / 88.3 = 0.47				
**Atom economy**	(180.132 / (166.223 + 31.999)) x 100 = 91%				
**Carbon economy**	100%				
**Reaction mass efficiency**	88.29 / (83.11 + 31.9) x100 = 77%				

Table S4. Green Chemistry Metrics calculations for 15 mg loading of the $Fe_{0.6}Mn_{0.4}(OH)_y$-*12S-6w* catalyst.

Catalyst Amount	10 mg	15 mg
**Yield %**	73	98
**E-factor**	0.90	0.47
**Atom economy %**	91	91
**Carbon economy %**	100	100
**Reaction mass efficiency %**	57	77

Table S5. Green chemistry metrics for different catalyst loadings of $Fe_{0.6}Mn_{0.4}(OH)_y$-*12S-6w*.

Sample Name	Path	N [a]	R (Å) [b]	σ^2 (x10 $^{-3}$ Å ^{2}) [c]	ΔE [d] (eV)	[e] R factor	Scattering
$Fe_{0.6}Mn_{0.4}(OH)_y$-*12S-6w*	Fe-O1	3.51	1.96	7.7	-1.05	0.0023	SS
$Fe_{0.6}Mn_{0.4}(OH)_y$-*12S-6w*	Fe-H1	4.20	2.88	7.7	-1.05	0.0023	SS
$Fe_{0.6}Mn_{0.4}(OH)_y$-*12S-6w*	Fe-Fe1	2.14	3.00	8.2	-1.05	0.0023	SS
$Fe_{0.6}Mn_{0.4}(OH)_y$-*12S-6w*	Fe-Fe2	7.30	3.49	12.5	-1.05	0.0023	SS
$Fe_{0.6}Mn_{0.4}(OH)_y$-*12S-6w*	Fe-O2	5.60	3.61	7.7	-1.05	0.0023	SS

[a]Coordination number (Degeneracy Number) and amplitude reduction factor (S_{0^2}) is calculated as 0.80 from the Fe foil and same value was used for all the fits. [b]Distance between absorbing atom and the atom in the corresponding shell. [c]Debye-Waller value. [d]The energy shift between fit and the experimental data. [e]R-factor shows the conformity level of the fit and measured data.

Table S6. EXAFS best fitting parameters for the Fe K-edge of $Fe_{0.6}Mn_{0.4}(OH)_y$-*12S-6w*.

Sample Name	Path	N [a]	R (Å) [b]	σ^2 (x10 $^{-3}$ Å ^{2}) [c]	ΔE [d] (eV)	[e] R factor	Scattering
$Fe_{0.6}Mn_{0.4}(OH)_y$-*12S-6w*	Mn-O1	2.7	1.92	5.5	-3.27	0.015	SS
$Fe_{0.6}Mn_{0.4}(OH)_y$-*12S-6w*	Mn-H1	4.58	2.45	4.8	-3.27	0.015	SS
$Fe_{0.6}Mn_{0.4}(OH)_y$-*12S-6w*	Mn-Mn1	2.46	2.96	7.9	-3.27	0.015	SS
$Fe_{0.6}Mn_{0.4}(OH)_y$-*12S-6w*	Mn-O-H2	9.69	2.70	4.8	-3.27	0.015	MS
$Fe_{0.6}Mn_{0.4}(OH)_y$-*12S-6w*	Mn-H3	4.29	2.72	4.8	-3.27	0.015	SS
$Fe_{0.6}Mn_{0.4}(OH)_y$-*12S-6w*	Mn-O2	4.58	3.43	0.2	-3.27	0.015	SS

[a]Coordination number (Degeneracy Number) and amplitude reduction factor (S_{0^2}) is calculated as 0.80 from the Mn foil and same value was used for all the fits. [b]Distance between absorbing atom and the atom in the corresponding shell. [c]Debye-Waller value. [d]The energy shift between fit and the experimental data. [e]R-factor shows the conformity level of the fit and measured data.

Table S7. EXAFS best fitting parameters for the Mn K-edge of $Fe_{0.6}Mn_{0.4}(OH)_y$-*12S-6w*.

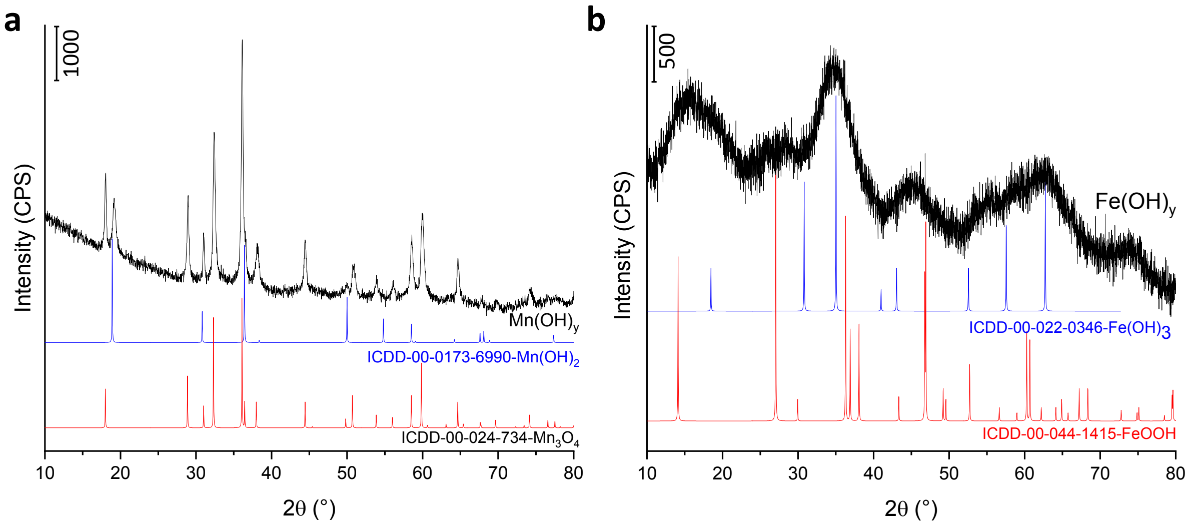

Figure S2. XRD data for a) $Mn(OH)_y$ and b) $Fe(OH)_y$ monometallic benchmark catalysts compared with reference cards from ICDD database. Catalysts were synthesized using a NaOH(aq) concentration of *12S*. (S represents a NaOH(aq) concentration of 2.06 M).

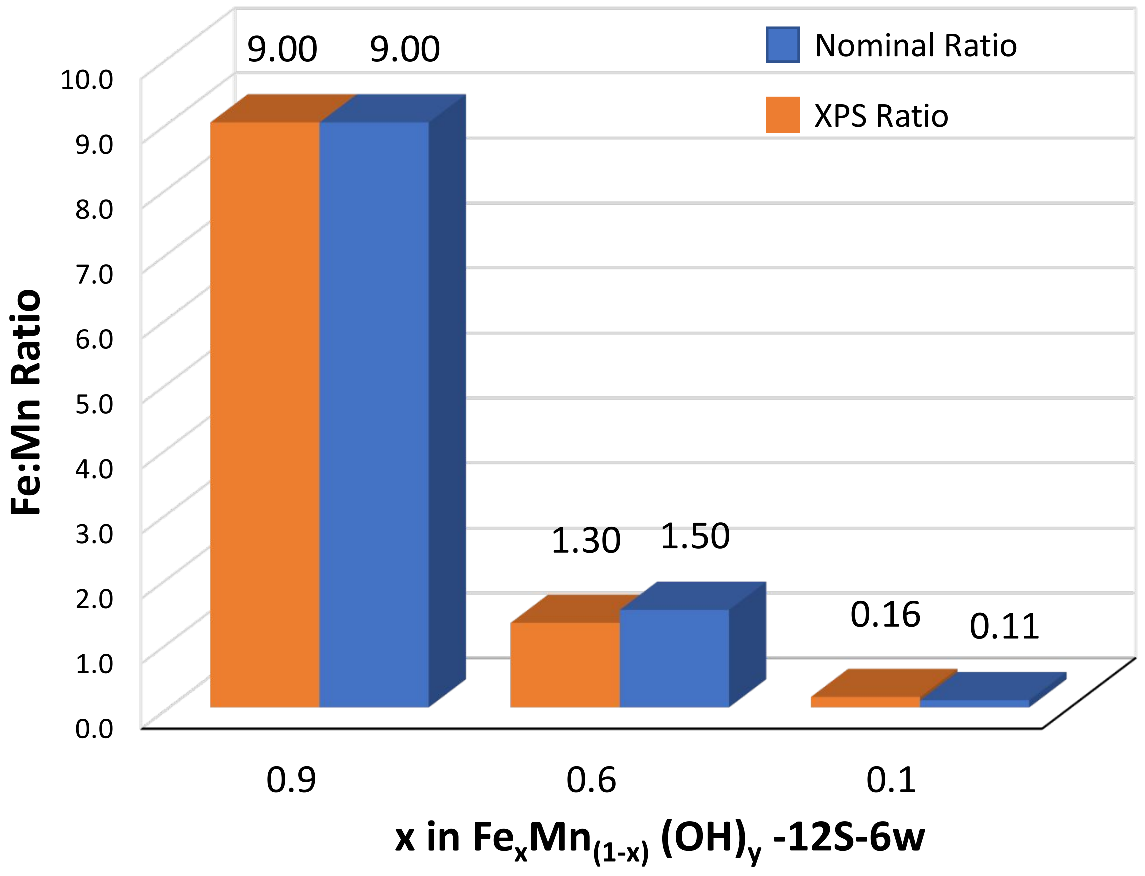

Figure S3. Comparison of Fe:Mn *surface* atomic ratios between nominal (represented by orange columns) and experimentally determined values obtained from XPS measurements (represented by blue columns) for $Fe_xMn_{(1-x)}(OH)_y$-*12S-6w* catalysts. *S* represents a NaOH(aq) concentration of 2.06 M.

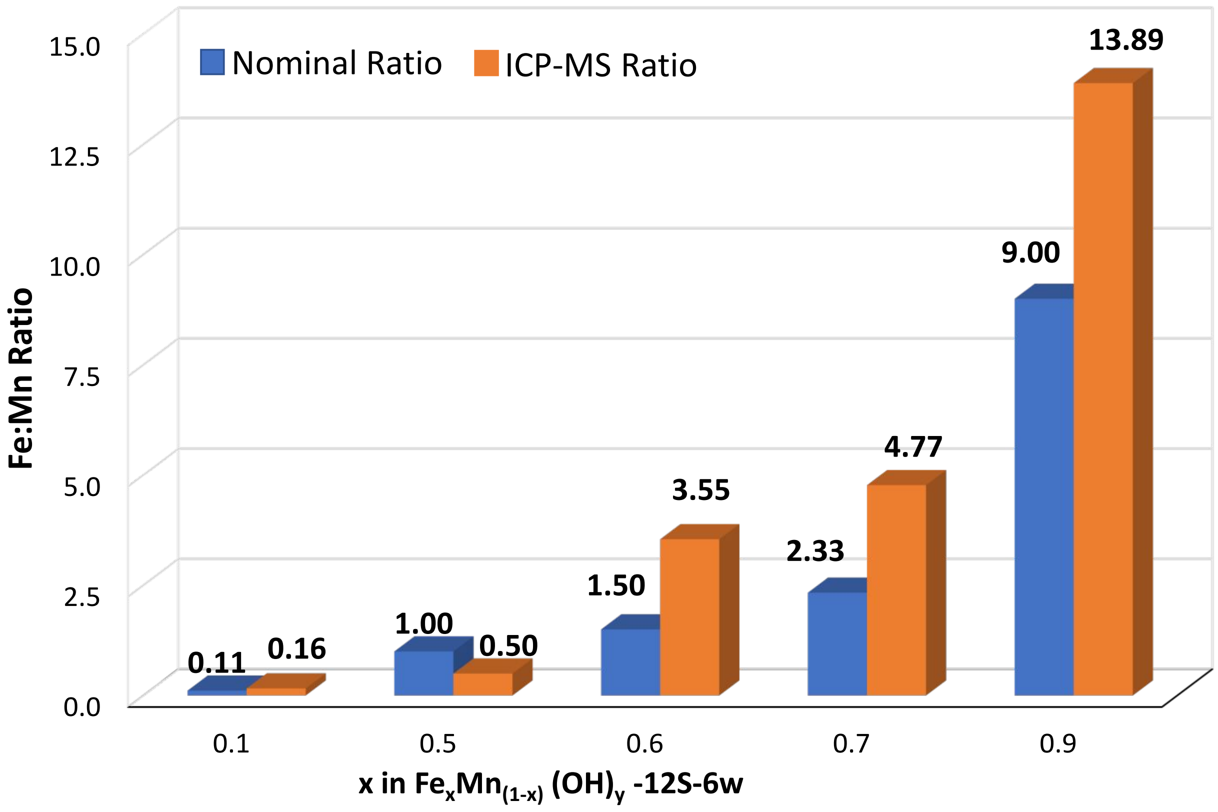

Figure S4. The Fe:Mn *bulk* atomic ratios determined via ICP-MS measurements for $Fe_xMn_{(1-x)}(OH)_y$-12S-6w catalysts (blue) as compared to the nominal Fe:Mn atomic ratios used in the synthesis (orange). *S* represents a NaOH(aq) concentration of 2.06 M.

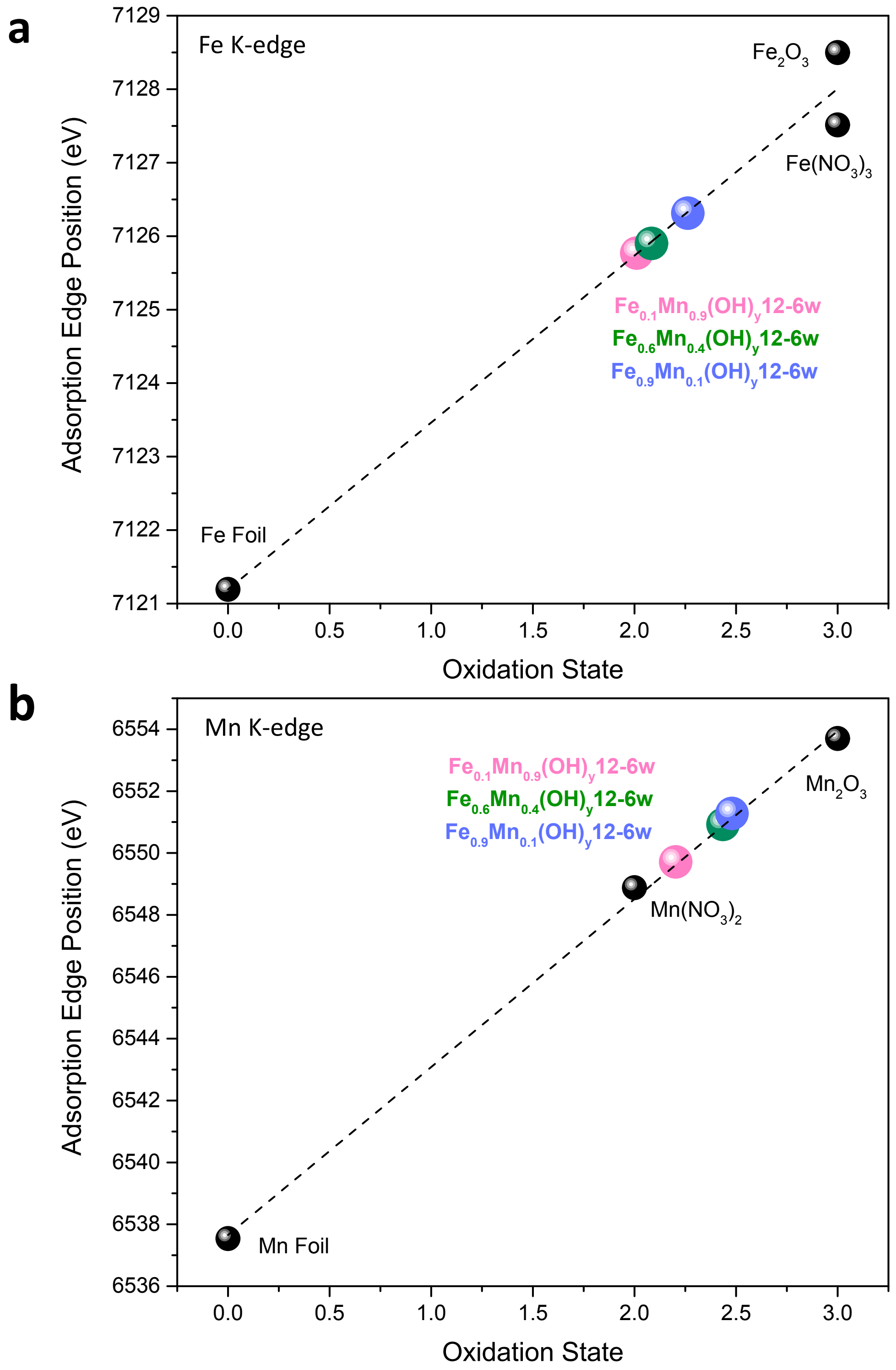

Figure S5. Average *bulk* (a) Fe and (b) Mn oxidation states of selected samples and reference materials as a function of corresponding K-edge positions determined via XANES experiments.

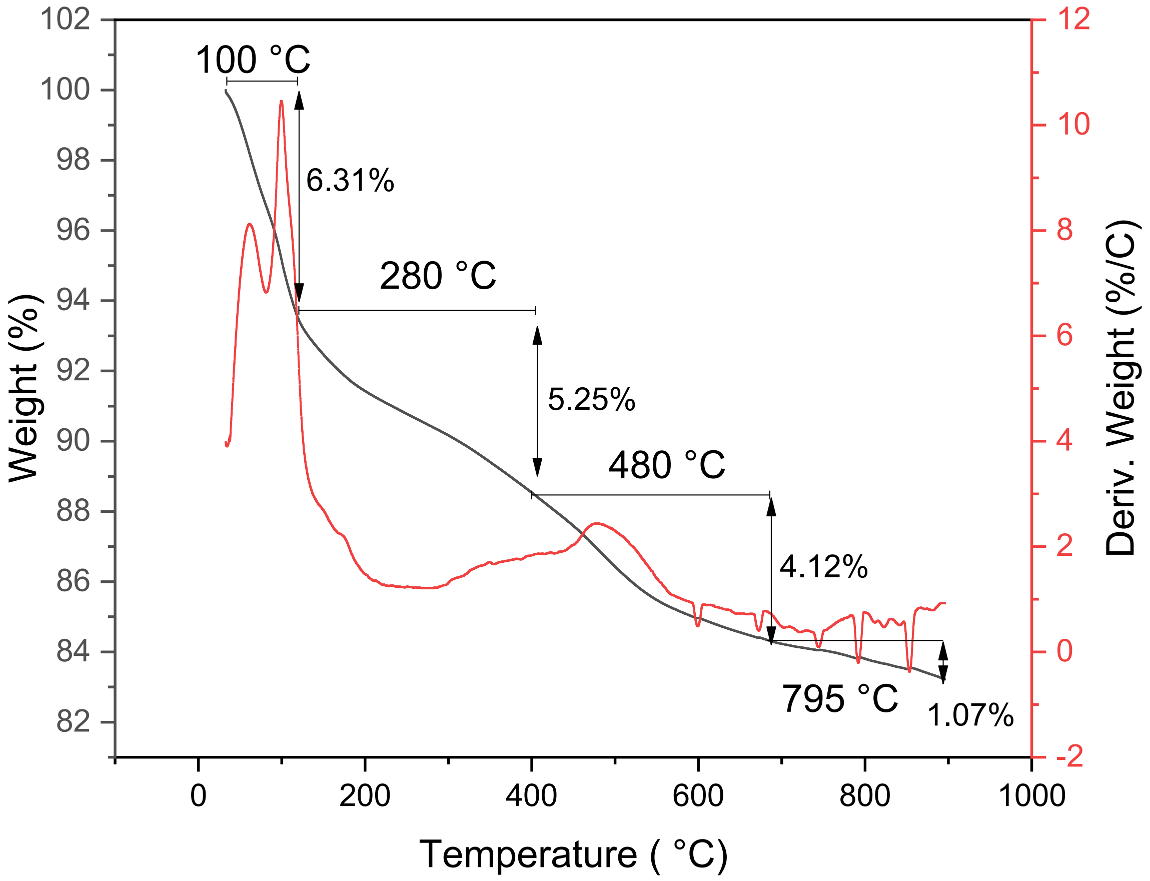

Figure S6. TGA analysis of the optimized $Fe_{0.6}Mn_{0.4}(OH)_y$-12S-6w catalyst.

TGA results indicate 4 major temperatures for weight loss: 6.3% weight loss at 100 °C, 5.3% weight loss at 280 °C, 4.1% weight loss at 480 °C, and 1.1% weight loss at 795 °C which can be explained in light of existing literature.[1,2] Weight loss events at 100 °C and 280 °C are likely to occur due to the removal of chemisorbed/physisorbed water molecules and the conversion of hydroxide structures to oxy-hydroxide structures, respectively. Weight loss at 480 °C can be associated with the completion of oxy-hydroxide structure formation and the onset of the dehydroxylation process. While the fourth weight loss event at 795 °C is likely due to the complete dehydroxylation of oxy-hydroxide structures into oxide structures. From these results, it can be readily inferred that the optimized $Fe_{0.6}Mn_{0.4}(OH)_y$-12S-6w catalyst can consistently catalyze reactions within 30-200 °C (i.e., temperature window utilized in the current work) without any significant loss in its structural integrity. As mentioned in the introduction of our manuscript, one of the major aims of the current work is to develop a catalyst to be utilized at

mild (low) temperatures, and our results clearly demonstrate that the optimized catalyst is indeed suitable for this application.

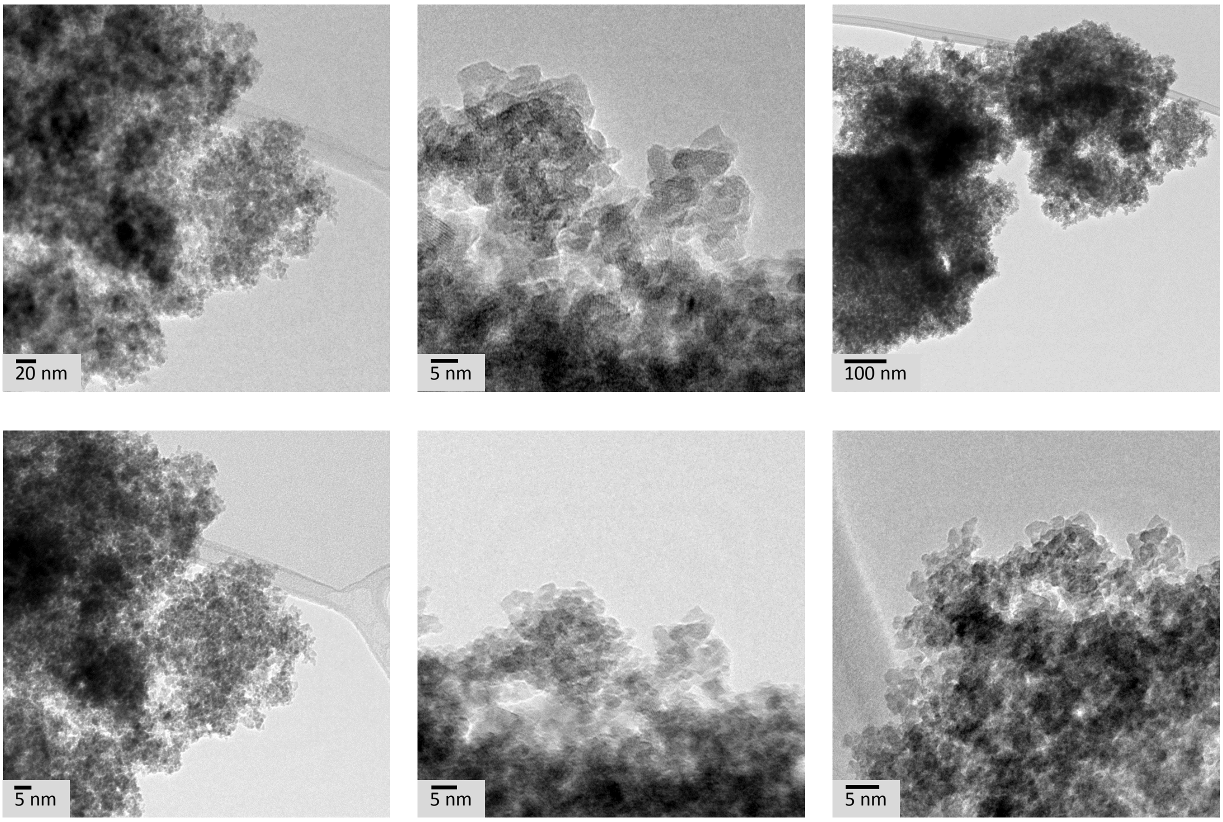

Figure S7. TEM images of the optimized $Fe_{0.6}Mn_{0.4}(OH)_y$-12S-6w catalyst.

Regeneration Protocol:

The regeneration protocol involved the following steps: the spent catalyst was washed three times with *12S* NaOH solution at 6000 rpm for 10 min. Subsequently, the catalyst underwent three additional washes using deionized (DI) water. Finally, the regenerated catalyst was dried at 60 °C for 12 h.

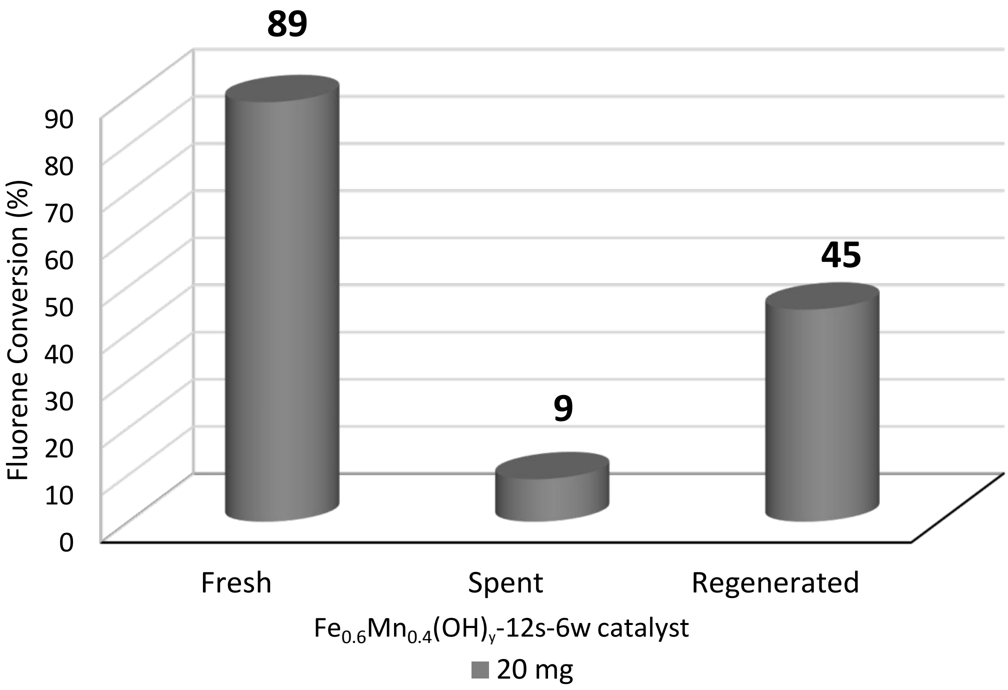

Figure S8. Catalytic activity data for $Fe_{0.6}Mn_{0.4}(OH)_y$-*12S-6w* in the aerobic oxidation of fluorene (1a) to fluorenone (1b) with a) fresh b) spent c) regenerated catalyst.

Experimental Procedures:

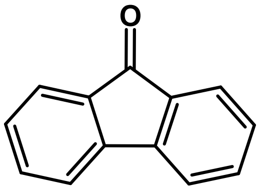

1b

9*H*-Fluoren-9-one (1b). An oven-dried 25 mL Schlenk tube was charged with fluorene (**1a**) (83.1 mg, 0.50 mmol), $Fe_{0.6}Mn_{0.4}(OH)_y$-*12S-6w* (15 mg) and *n*-heptane (2.0 mL). The Schlenk tube was filled with O_2 gas (1 bar) and sealed. The reaction mixture was stirred at 90 °C in an oil bath for 24 h. After the reaction mixture was cooled to room temperature, it was diluted with EtOAc, and passed through a small pad of Celite. Pure fluorenone (**1b**) (88.5 mg, 98% yield) was obtained as a yellow solid after purification by flash column chromatography (SiO_2,

EtOAc/hexanes = 1:9). The spectral data are in agreement with the reported values in the literature.[3]

^{1}H NMR (400 MHz, CDCl$_3$) δ: 7.66-7.64 (2H, m), 7.52-7.45 (4H, m), 7.28 (2H, td, J = 7.2, 1.4 Hz).

^{13}C NMR (100 MHz, CDCl$_3$) δ: 194.0, 144.6, 134.8, 134.3, 129.2, 124.4, 120.4.

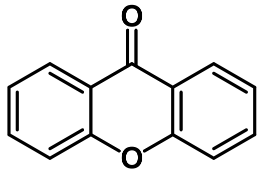

2b

9*H*-Xanthen-9-one (2b). An oven-dried 25 mL Schlenk tube was charged with xanthene (**2a**) (91.1 mg, 0.50 mmol), $Fe_{0.6}Mn_{0.4}(OH)_y$-*12S-6w* (15 mg), and *n*-octane (2.0 mL). The Schlenk tube was filled with O_2 gas (1 bar) and sealed. The reaction mixture was stirred at 110 °C in an oil bath for 24 h. After the reaction mixture was cooled to room temperature, it was diluted with EtOAc, and passed through a small pad of Celite. Pure xanthone (**2b**) (90.1 mg, 92% yield) was obtained as a light-brown solid after purification by flash column chromatography (SiO_2, hexanes then EtOAc/hexanes = 1:9). The spectral data are in agreement with the reported values in the literature.[4]

^{1}H NMR (400 MHz, CDCl$_3$) δ: 8.36 (2H, dd, J = 8.0, 1.8 Hz), 7.74 (2H, ddd, J = 8.8, 7.0, 1.8 Hz), 7.51 (2H, dd, J = 8.5, 1.0 Hz), 7.39 (2H, ddd, J = 8.0, 7.0, 1.3 Hz).

^{13}C NMR (100 MHz, CDCl$_3$) δ: 177.4, 156.4, 135.0, 126.9, 124.1, 122.1, 118.1.

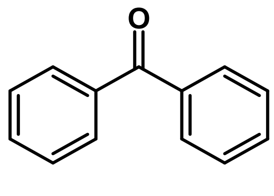

4b

Benzophenone (4b). An oven-dried 25 mL Schlenk tube was charged with diphenylmethane (**4a**) (42.1 mg, 0.25 mmol), $Fe_{0.6}Mn_{0.4}(OH)_y$-*12S-6w* (37 mg), and chlorobenzene (1.0 mL). The Schlenk tube was filled with O_2 gas (1 bar) and sealed. The reaction mixture was stirred at 130 °C in an oil bath for 24 h. After it was cooled to room temperature, it was diluted with EtOAc, and passed through a small pad of Celite. Pure benzophenone (**4b**) (39.6 mg, 87% yield) was obtained as a colorless oil after purification by flash column chromatography (SiO_2, hexanes only). The spectral data are in agreement with the reported values in the literature.[4]

^{1}H NMR (400 MHz, $CDCl_3$) δ: 7.81 (4H, dd, *J* = 8.3, 1.5 Hz), 7.59 (2H, tt, *J* = 7.5, 1.4 Hz), 7.48 (4H, app t, *J* = 7.5 Hz).

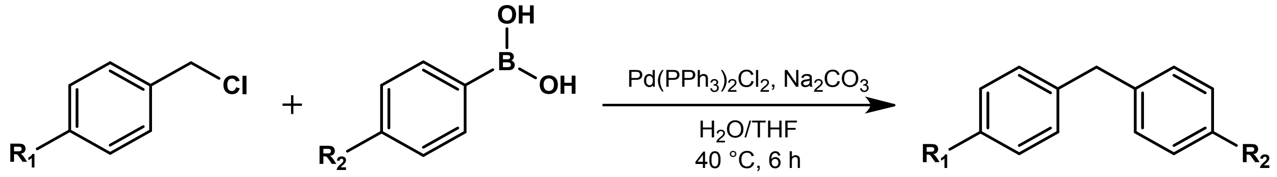

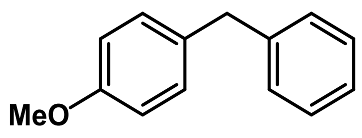

5a

4-Methoxydiphenylmethane (5a). This compound was prepared following a reported literature procedure.[5] An oven-dried, 25 mL Schlenk tube was subjected to vacuum-nitrogen cycle three times. $Pd(PPh_3)Cl_2$ (0.015 mmol, 10.5 mg) and Na_2CO_3 (2.68 mmol, 284 mg) were

dissolved in 1 mL of THF and 1 mL of distilled H_2O. Then phenylboronic acid (1.34 mmol, 183 mg) and 4-methoxybenzyl chloride (1.07 mmol, 169 mg) were added to the reaction mixture, sequentially. The walls of the Schlenk tube were washed with 0.5 mL of THF and 0.5 mL of H_2O. The resulting reaction mixture was stirred in an oil bath at 40 °C for 6 h with a condenser. The reaction mixture was cooled to room temperature and quenched with distilled water. Then the aqueous phase was extracted three times with CH_2Cl_2. The combined organic layers were dried over Na_2SO_4, filtered and concentrated under reduced pressure. Purification by flash column chromatography (hexanes only) gave 4-methoxydiphenylmethane (**5a**) as a white solid (145 mg, 68% yield). The spectral data are in agreement with the reported values in the literature.[6]

^{1}H NMR (400 MHz, CDCl$_3$) δ: 7.24 (2H, t, *J* = 7.4 Hz), 7.19-7.11 (3H, m), 7.07 (2H, d, *J* = 8.8 Hz), 6.79 (2H, d, *J* = 8.5 Hz), 3.88 (2H, s), 3.70 (3H, s).

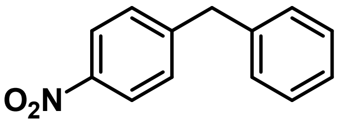

6a

4-Nitrodiphenylmethane (6a). This compound was prepared following a reported literature procedure.[3] An oven-dried, 25 mL Schlenk tube was subjected to vacuum-nitrogen cycle three times. $Pd(PPh_3)Cl_2$ (0.024 mmol, 16.6 mg) and Na_2CO_3 (3.95 mmol, 419 mg) were dissolved in 1 mL of THF and 1 mL of distilled H_2O. Then 4-nitrophenylboronic acid (1.98 mmol, 330 mg) and benzyl chloride (1.58 mmol, 196 mg) were added to the reaction mixture sequentially. The walls of the flask were washed with 0.5 mL of THF and 0.5 mL of H_2O. The resulting reaction mixture was stirred in an oil bath at 40 °C for 6 h with a condenser. The reaction mixture was cooled to room temperature and quenched with distilled water. Then the aqueous phase was extracted three times with CH_2Cl_2. The combined organic layers were dried over

Na_2SO_4, filtered and concentrated under reduced pressure. Purification by flash column chromatography (hexanes only) gave 4-nitrodiphenylmethane (**6a**) as a white solid (284 mg, 84% yield). The spectral data are in agreement with the reported values in the literature.[7]

^{1}H NMR (400 MHz, $CDCl_3$) δ: 8.09 (2H, d, *J* = 8.8 Hz), 7.38-7.28 (5H, m), 7.24 (2H, d, *J* = 7.0 Hz), 4.07 (2H, s).

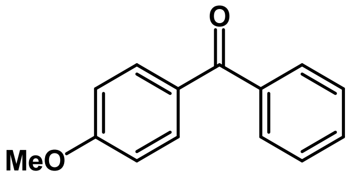

5b

4-Methoxybenzophenone (5b). An oven-dried 25 mL Schlenk tube was charged with 4-methoxydiphenylmethane (**5a**) (49.6 mg, 0.25 mmol), $Fe_{0.6}Mn_{0.4}(OH)_y$-*12S-6w* (37 mg) and chlorobenzene (1.0 mL). The Schlenk tube was filled with O_2 gas (1 bar) and sealed. The reaction mixture was stirred at 130 °C in an oil bath for 24 h. After the reaction mixture was cooled to room temperature, it was diluted with EtOAc, and passed through a small pad of Celite. Pure 4-methoxybenzophenone (**5b**) (31.3 mg, 59% yield) was obtained as a white solid after purification by flash column chromatography (SiO_2, hexanes only). The spectral data are in agreement with the reported values in the literature.[8]

^{1}H NMR (400 MHz, $CDCl_3$) δ: 7.83 (2H, d, *J* = 8.8 Hz), 7.75 (2H, d, *J* = 7.0 Hz), 7.56 (1H, t, *J* = 7.4 Hz), 7.47 (2H, t, *J* = 7.8 Hz), 6.96 (2H, d, *J* = 8.9 Hz), 3.88 (3H, s).

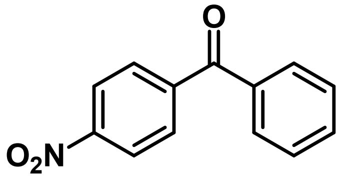

6b

4-Nitrobenzophenone (6b). An oven-dried 25 mL Schlenk tube was charged with 4-nitrodiphenylmethane (**6a**) (53.3 mg, 0.25 mmol), $Fe_{0.6}Mn_{0.4}(OH)_y$-*12S-6w* (37 mg), and chlorobenzene (1.0 mL). The Schlenk tube was filled with O_2 gas (1 bar) and sealed. The reaction mixture was stirred at 130 °C in an oil bath for 24 h. After it was cooled to room temperature, it was diluted with EtOAc, and passed through a small pad of Celite. Pure 4-nitrobenzophenone (**6b**) (50 mg, 93% yield) was obtained as a light-yellow solid after purification by flash column chromatography (SiO_2, hexanes only). The spectral data are in agreement with the reported values in the literature.[8]

^{1}H NMR (400 MHz, $CDCl_3$) δ: 8.34 (2H, d, J = 8.8 Hz), 7.93 (2H, d, J = 8.8 Hz), 7.80 (2H, d, J = 8.0 Hz), 7.65 (1H, t, J = 7.4 Hz), 7.52 (2H, t, J = 7.7 Hz).

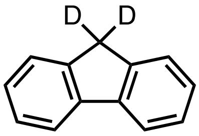

8

Compound **8** to be used in the KIE experiment was synthesized following the procedure reported in our previous work.[9]

NMR Spectra:

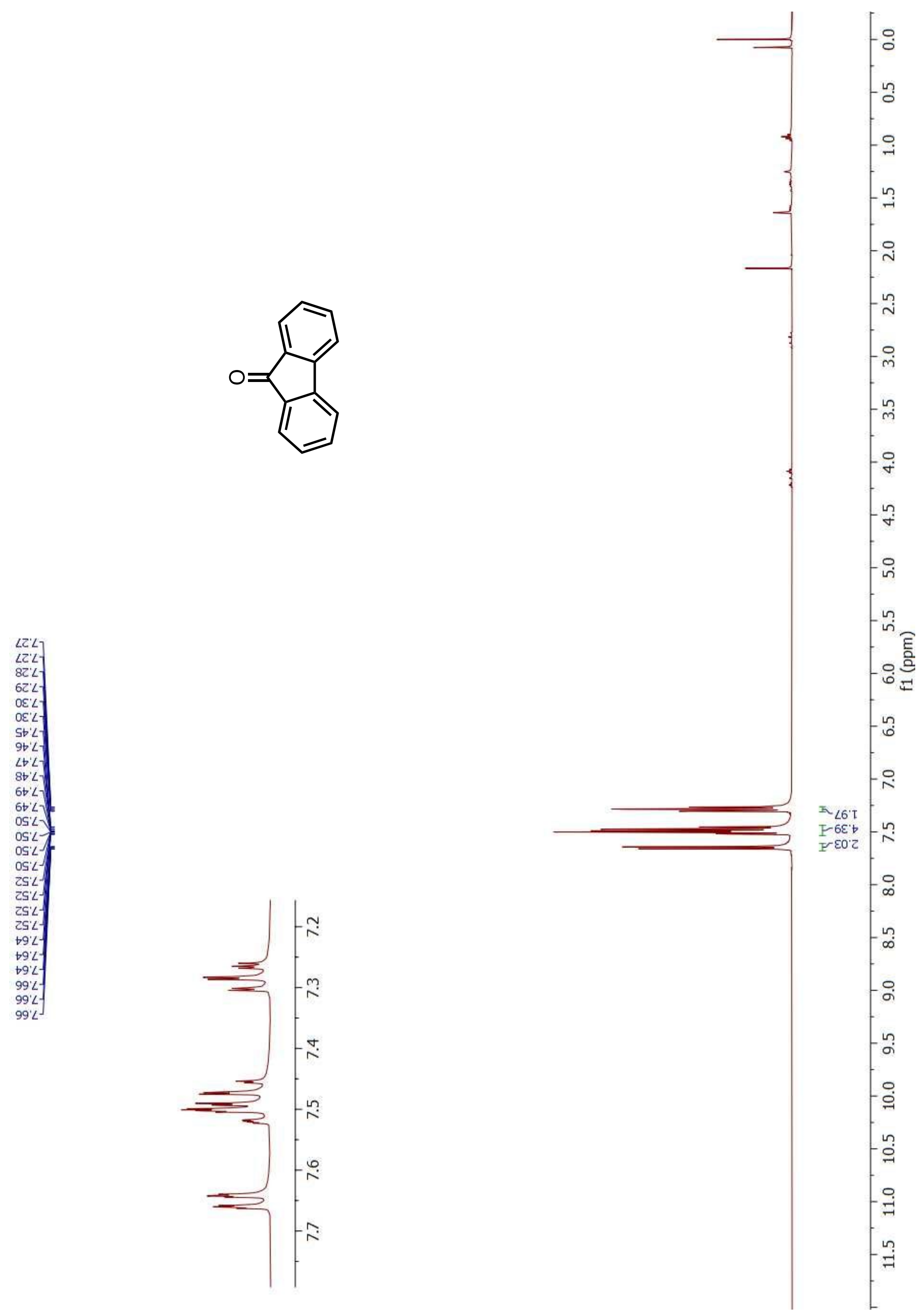

Figure S9. ^{1}H NMR spectrum of **1b** in $CDCl_3$.

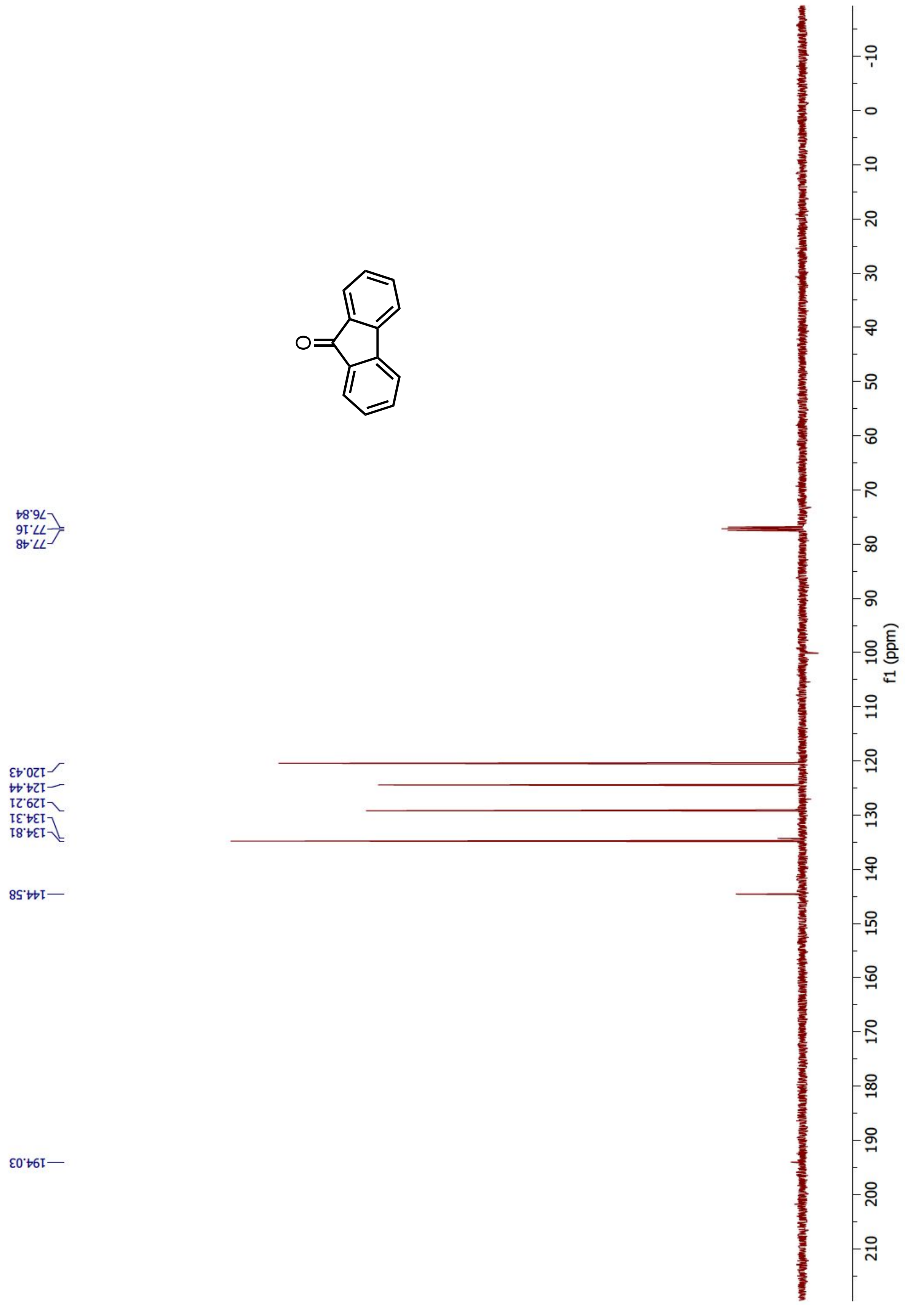

Figure S10. $^{13}C\{^{1}H\}$ NMR spectrum of **1b** in $CDCl_3$.

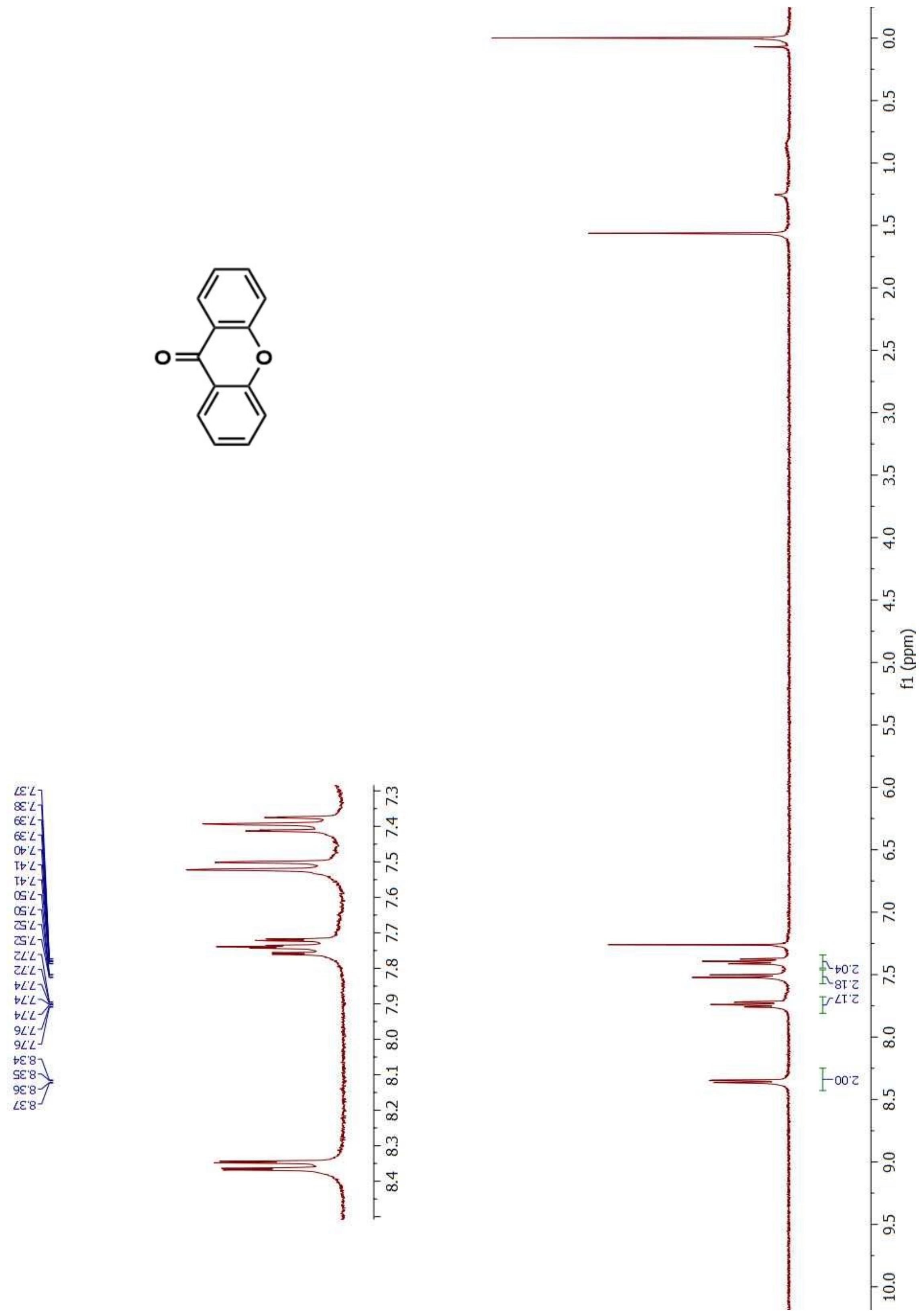

Figure S11. ^{1}H NMR spectrum of **2b** in $CDCl_3$.

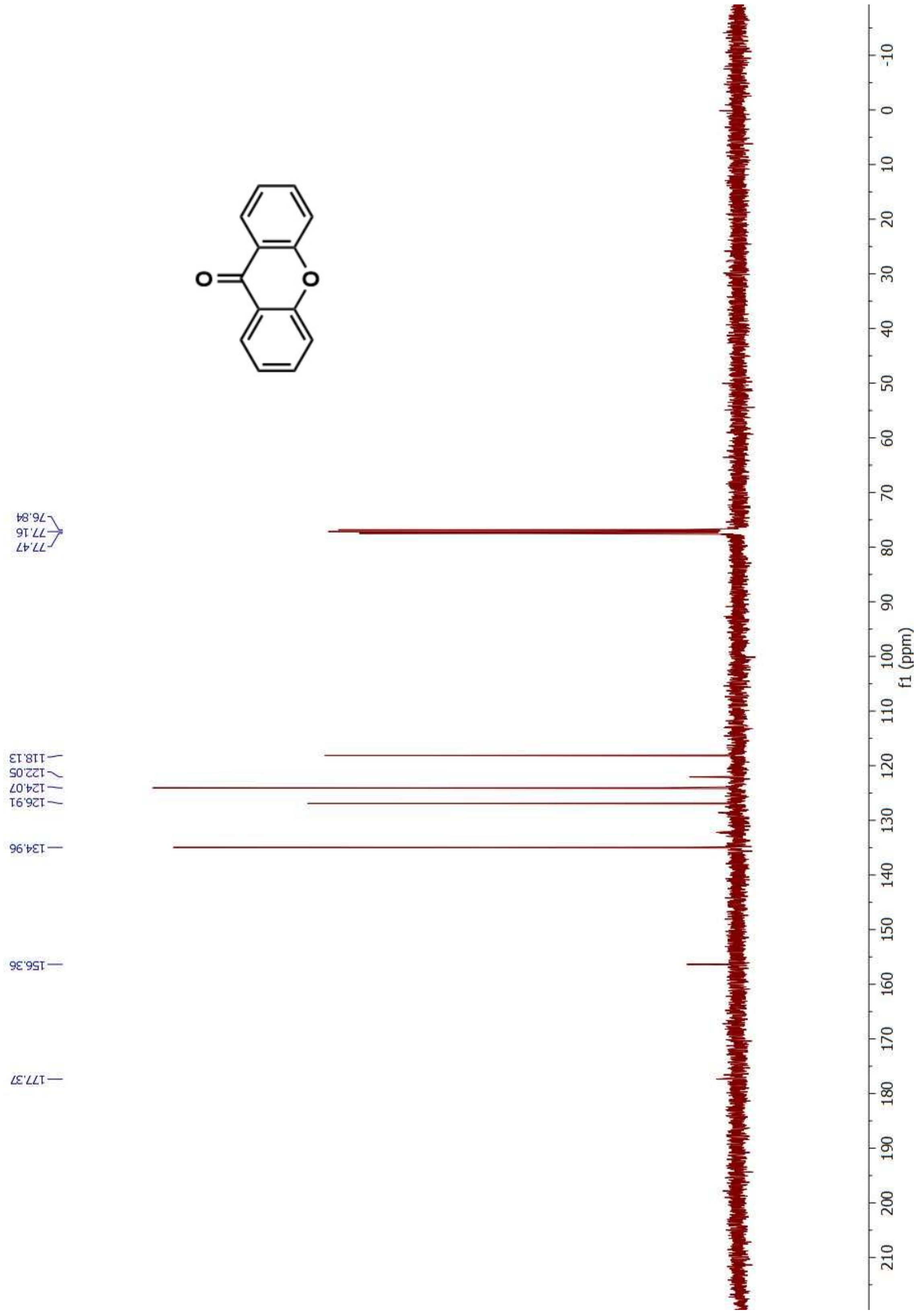

Figure S12. $^{13}C\{^{1}H\}$ NMR spectrum of **2b** in $CDCl_3$.

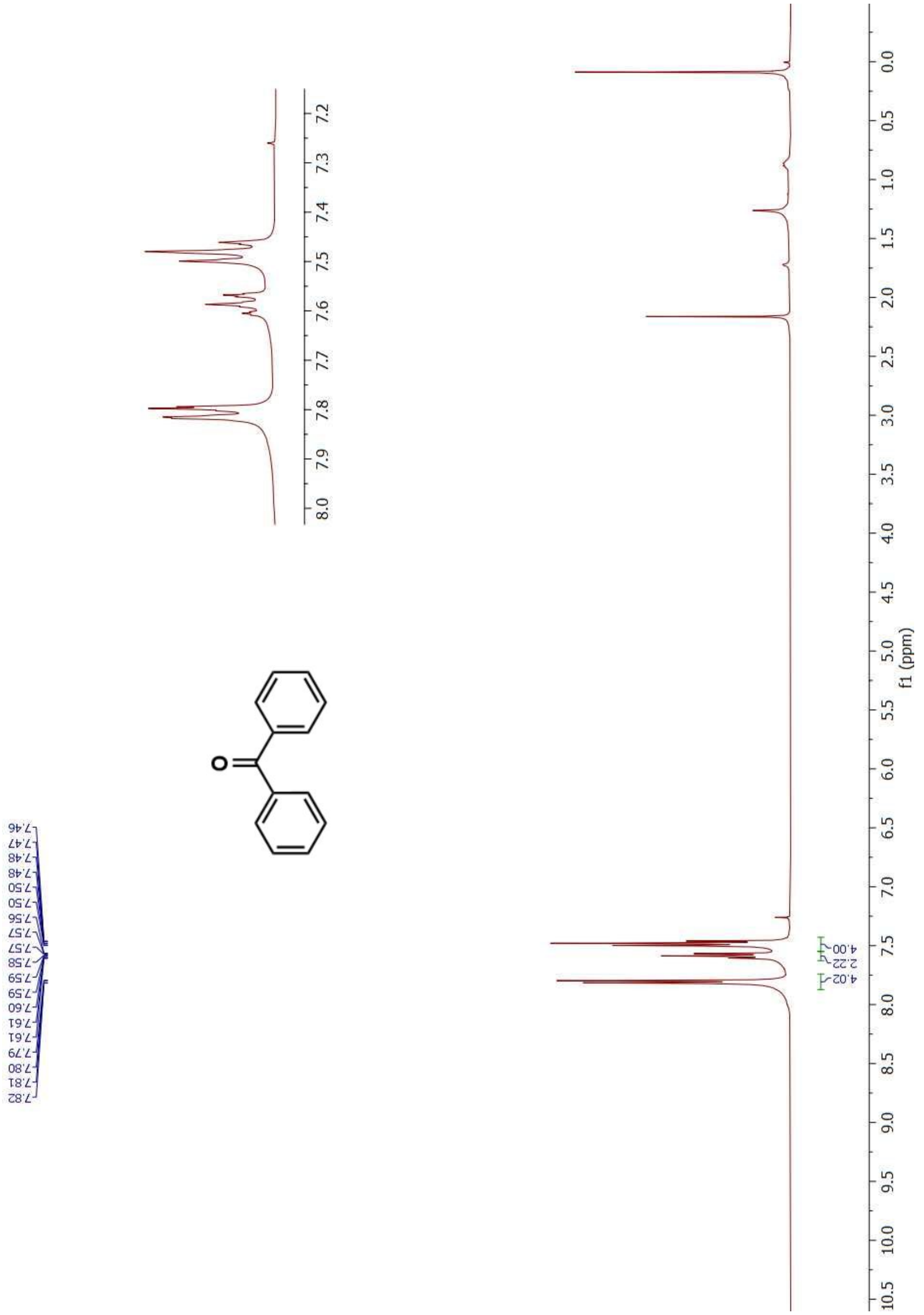

Figure S13. ^{1}H NMR spectrum of **4b** in $CDCl_3$.

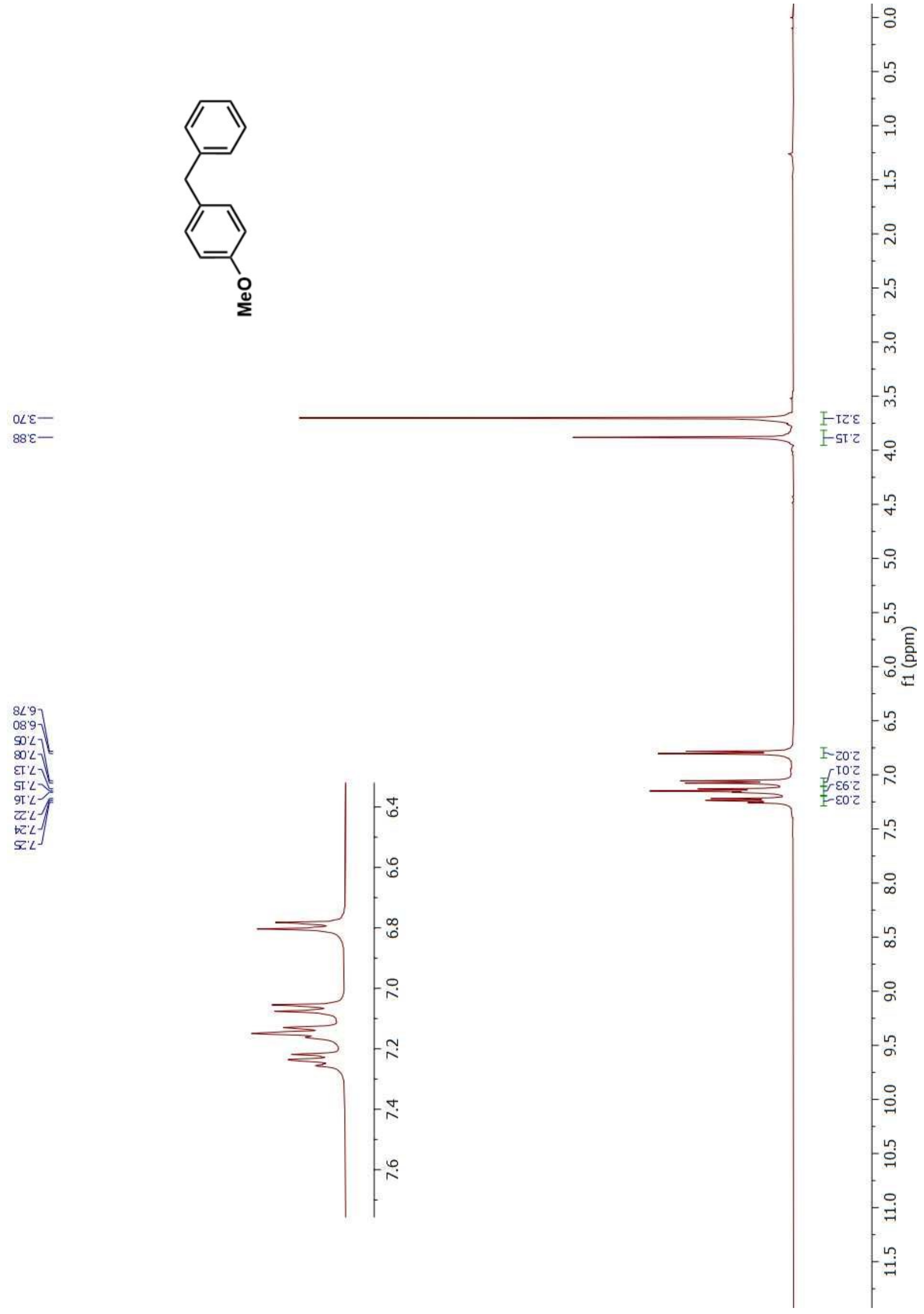

Figure S14. ^{1}H NMR spectrum of **5a** in $CDCl_3$.

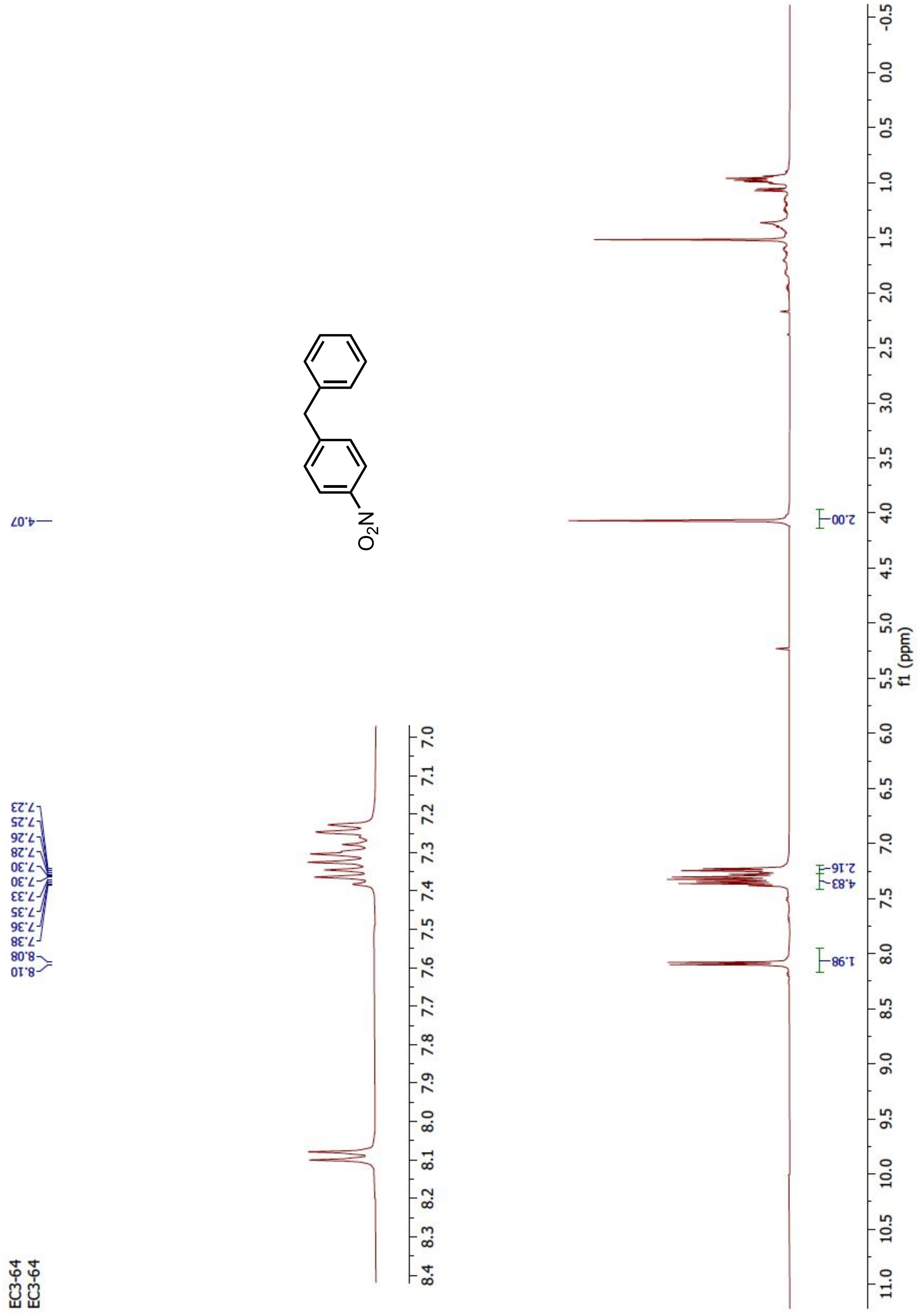

Figure S15. ^{1}H NMR spectrum of **6a** in $CDCl_3$.

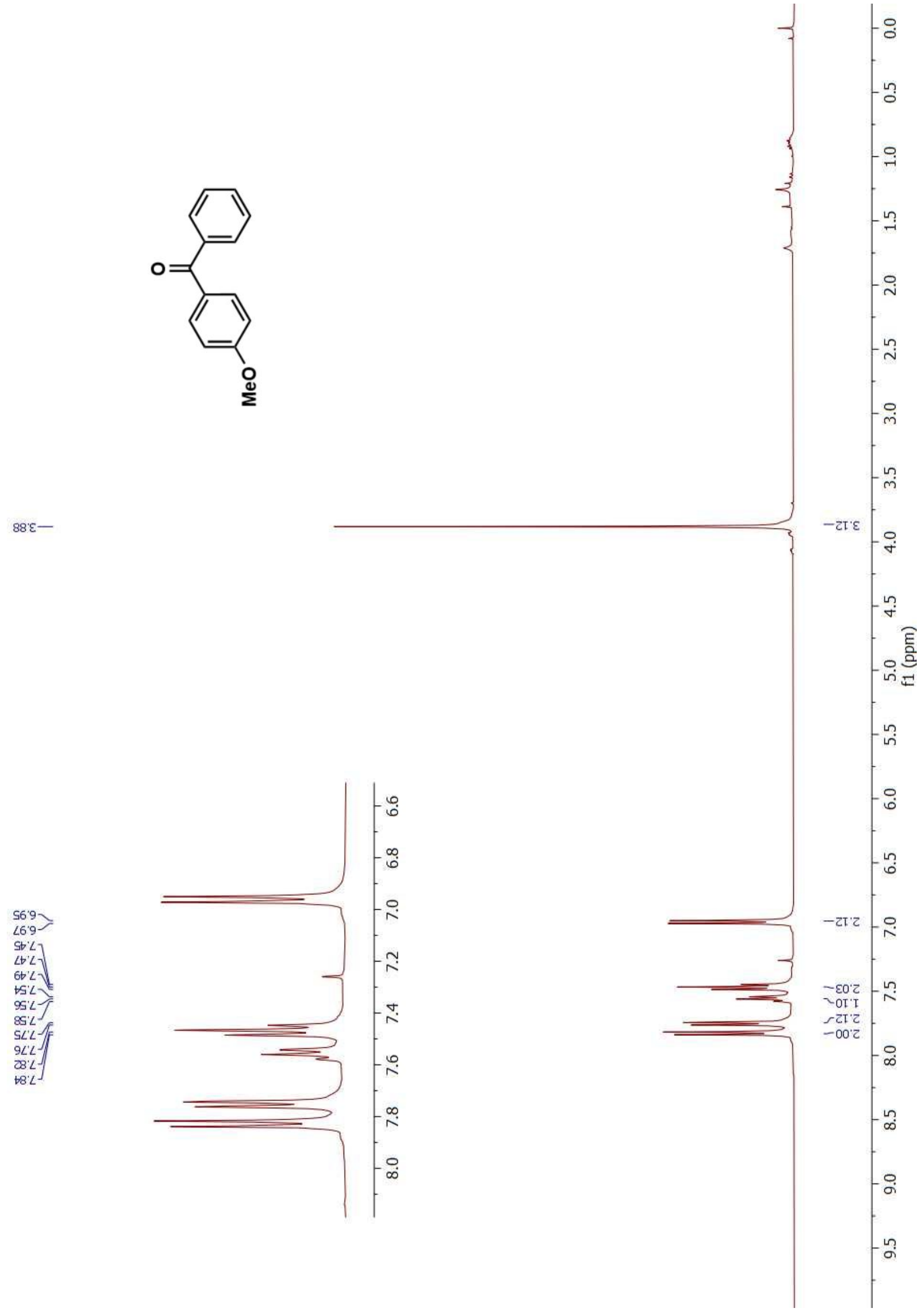

Figure S16. ^{1}H NMR spectrum of **5b** in $CDCl_3$.

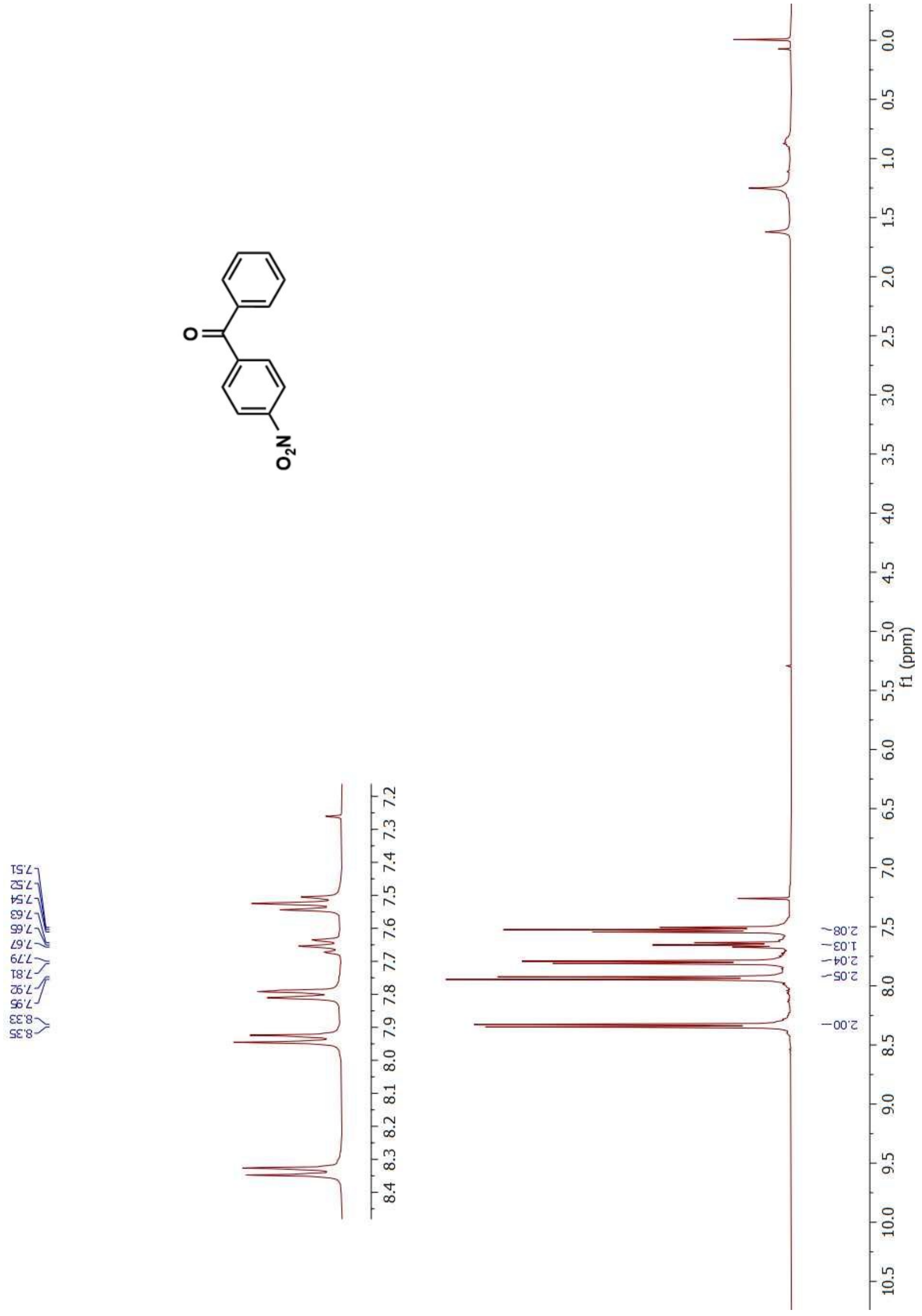

Figure S17. ^{1}H NMR spectrum of **6b** in $CDCl_3$.

References:

1. Bumajdad, A.; Ali, S.; Mathew, A. *J. Colloid Interface Sci.* **2011**, *355* (2), 282–292.
2. Parveen, M. F.; Umapathy, S.; Dhanalakshmi, V.; Anbarasan, R. *Journal of Applied Polymer Science* **2010.**
3. Seo, S.; Slater, M.; Greaney, M. F. *Org. Lett.* **2012**, *14*, 2650-2653.
4. Xiang, M.; Xin, Z.-K.; Chen, B.; Tung, C.-H.; Wu, L.-Z. *Org. Lett.* **2017**, *19*, 3009.
5. Ohsumi, M.; Ito, A.; Nishiwaki, N. *RSC Adv.* **2018**, *8*, 35056.
6. Dan, X.; Xing, L.; Tang, Y.; Wang, W.; Cai, Y. *Org. Lett.* **2023**, *25*, 4124.
7. Balaraman, K.; Wolf, C. *Org. Lett.* **2021**, *23*, 8994.
8. Wu, H.; Sumita, A.; Otani, Y.; Ohwada, T. *J. Org. Chem.* **2022**, *87*, 15224.
9. Sika-Nartey, A.; Sahin, Y.; Ercan, K. E.; Kap, Z.; Kocak, Y.; Erdali, A. D.; Erdivan, B.; Türkmen, Y. E.; Ozensoy, E. *ACS Appl. Nano Mater.* **2022**, *5*, 18855.
